# New 9α-Hydroxy-5α,6α-epoxysterols from the Vietnamese Marine Sponge *Ircinia echinata*

**DOI:** 10.3390/md16110424

**Published:** 2018-11-01

**Authors:** Thi Thanh Van Trinh, Bich Ngan Truong, Arlette Longeon, Thi Mai Huong Doan, Alexandre Deville, Van Minh Chau, Van Cuong Pham, Marie-Lise Bourguet-Kondracki

**Affiliations:** 1 Advanced Center for Bioorganic Chemistry of the Institute of Marine Biochemistry, Vietnam Academy of Science and Technology, 18 Hoang Quoc Viet, Caugiay 8424, Hanoi, Vietnam; trtvan76@yahoo.com (T.T.V.T.); tbngan1977@yahoo.com (B.N.T.); doanhuong7@yahoo.com (T.M.H.D.); cvminh@vast.vn (V.M.C.); 2 Molécules de Communication et Adaptation des Microorganismes, UMR 7245 CNRS, Muséum National d’Histoire Naturelle, 57 rue Cuvier (CP54), 75005 Paris, France; arlette.longeon@mnhn.fr (A.L.); alexandre.deville@mnhn.fr (A.D.)

**Keywords:** marine sponge, *Ircinia echinata*, 9α-hydroxy-5α,6α-epoxysterols, cytotoxicity

## Abstract

Chemical investigation of the methanol extract of the Vietnamese marine sponge *Ircinia echinata* led to the isolation of six new 9α-hydroxy-5α,6α-epoxysterols: 5α,6α-epoxycholesta-7,22(*E*)-dien-3β,9α-diol (**1**), 5α,6α-epoxycholesta-7,24(28)-dien-3β,9α-diol (**2**), (24*R*)-5α,6α-epoxy-24-ethyl-cholesta-7-en-3β,9α-diol (**3**), 5α,6α-epoxycholesta-7-en-3β,9α-diol (**4**), (24*S*)-5α,6α-epoxyergosta-7,22-dien-3β,9α-diol (**5**), and (24*R*)-5α,6α-epoxy-24-methyl-cholesta-7-en-3β,9α-diol (**6**) along with the known 5α-6α-epoxysterols: 5α,6α-epoxystigmasta-7-en-3β-ol (**7**), 5α,6α-epoxystigmasta-7,22-dien-3β-ol (**8**), and 5α,6α-epoxyergosta-7-en-3β-ol (**9**). Their structures and their configurations were established on the basis of high resolution mass spectra and extensive 1D and 2D NMR spectroscopic data and by comparison with the literature. Their cytotoxic activity, evaluated against three human cancer cell lines, MCF-7, Hep-G2 and LU-1, revealed that only compounds **3** and **4** exhibited significant antiproliferative activity and compound **3** showed a selective inhibition towards the MCF-7 human breast cancer cells.

## 1. Introduction

Marine sponges of the genus *Ircinia* are known as a rich source of varied bioactive natural products, including fatty acids [1], steroids [2,3], sesqui- [4] and sester-terpenes [5,6,7], peptides [8], and macrolides [9,10]. Previous reports revealed that steroids from marine sponges of the genus *Ircinia* exhibited a wide range of biological activities, as illustrated with the antileishmanial epidioxysterols from the Colombian marine sponge *Ircinia campana* [11], the cytotoxic 5,6-epoxysterols from the Chinese sponge *Ircinia aruensis* [12], the cytotoxic pentacyclic steroids possessing a cis C/D ring junction from the Okinawan marine sponge *Ircinia* sp. [13], the antibacterial 9,11-secosterol [14], or the derivative linked with a 1,4-quinone from a Korean marine sponge *Ircinia* sp. [15].

The East Sea in Vietnam covers an area of approximately three million km^2^, and has about 3000 km of coastline. The marine biodiversity of the East Sea is considered to be among the most extensive in the world, but remains poorly understood and explored. Recently, Kiem *et al.* reported the isolation of several furanosesterterpenes and six known sterols from the sponge *Ircinia echinata* collected at Co To, Quang Ninh, Vietnam [16,17]. In this paper, we describe the isolation and structural characterization of six new 9α-hydroxy-5α,6α-epoxysterols (**1**–**6**), together with three known 5α,6α-epoxysterols from *I. echinata* (Order Dictyoceratida, Family Irciniidae) collected at Bai Tu Long, Vietnam. The cytotoxic activity of the nine 5α,6α-epoxysterols was evaluated against different human tumor cell lines.

## 2. Results and Discussion

**Figure 1:**
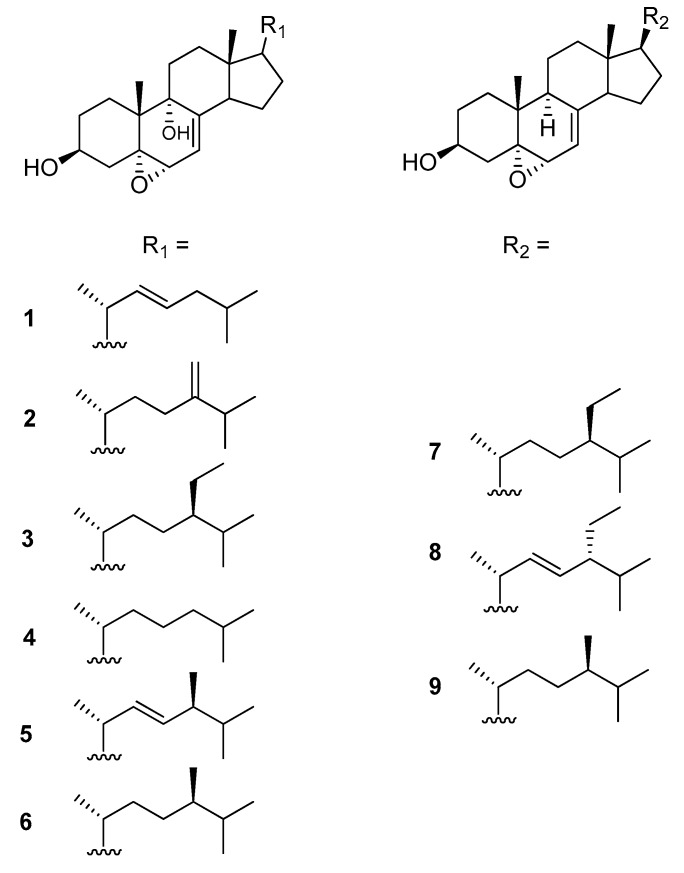
Structure of 5α,6α-epoxysterols **1**–**9** isolated from *Ircinia echinata*.

Dried materials of *I. echinata* (0.5 kg) were extracted with MeOH at room temperature. The MeOH-soluble product was purified by a flash chromatography silica column followed by silica gel, Sephadex LH-20, and repeated high performance liquid chromatography (HPLC) columns to yield compounds **1**–**9** (Figure 1).

**Figure 2:**
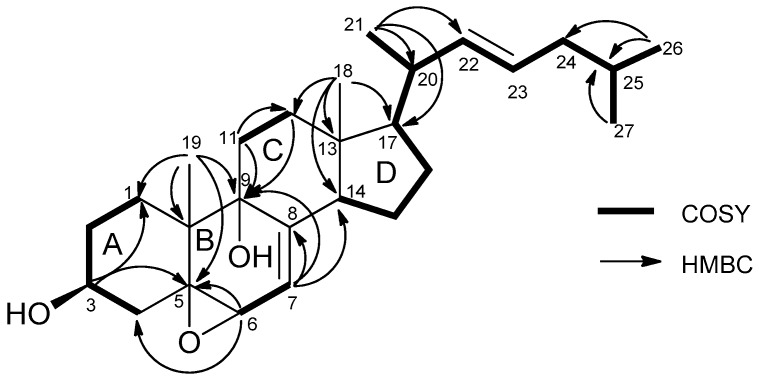
Selected HMBC and COSY correlations in compound **1**.

Compound **1** was isolated as white microcrystals. The HRESIMS exhibited a fragment ion peak at *m*/*z* 379.2953 [M-2H_2_O + H]^+^ (calculated 379.3001 for C_27_H_39_O), corresponding for compound **1** to a molecular formula of C_27_H_42_O_3_. Seven degrees of unsaturation was thus deduced for **1**. The presence of a cholestanol skeleton was suggested by the ^1^H nuclear magnetic resonance (NMR) spectrum (Table 1), which exhibited two singlet methyls at δ_H_ 0.66 (CH_3_-18) and 1.12 (CH_3_-19), three doublet methyls at δ_H_ 1.03 (*J* = 6.5 Hz, CH_3_-21) and 0.89 (*J* = 6.7 Hz, CH_3_-26 and CH_3_-27), and one oxygenated methine proton at δ_H_ 4.0 (m, H-3). Additionally, a set of complex protons was noted for the aliphatic region, including two olefinic protons at δ_H_ 5.32 (m, H-23) and 5.24 (dd, *J* = 8.0 and 15.0 Hz, H-22). The ^13^C NMR spectrum of compound **1** confirmed the presence of 27 carbons, including five methyls, eight methylenes, six sp^3^ methines, four sp^3^ quaternary carbons, and one sp^2^ quaternary carbon (Table 2). The chemical shifts of carbons at δ_C_ 68.2 (C-3), 78.9 (C-5), 73.7 (C-6), and 76.1 (C-9) suggested their linkage to oxygen. Analysis of the homonuclear correlated spectroscopy (COSY) spectrum of **1** revealed the structural elements, as presented in Figure 2. The connections of these spin–spin coupling systems were also shown from heteronuclear multiple bond correlations (HMBC) that confirmed the cholestanol skeleton for **1**. Therefore, correlations from the methyl protons at δ_H_ 1.12 (CH_3_-19) with the carbons at δ_C_ 28.2 (C-1), 78.9 (C-5), 76.1 (C-9), and 41.4 (C-10), and from the multiplet proton at δ_H_ 4.0 (H-3) with the carbons at δ_C_ 78.9 (C-5) and 28.2 (C-1) assigned the presence of the A-ring. Similarly, the HMBC correlations from the proton at δ_H_ 3.65 (H-6) with the carbons at δ_C_ 78.9 (C-5) and 40.7 (C-4) as well as the correlations from the methyl protons at δ_H_ 1.12 (CH_3_-19) with the carbons at δ_C_ 78.9 (C-5), 76.1 (C-9), 41.4 (C-10), and 28.2 (C-1) established the B-ring. Finally, the C/D ring system was determined by HMBC correlations from the methylene protons at δ_H_ 1.60–1.91 (CH_2_-11), with the carbons at δ_C_ 76.1 (C-9) and 36.5 (C-12), and from the methyl protons at δ_H_ 0.66 (CH_3_-18), with the carbons at δ_C_ 36.5 (C-12), 44.8 (C-13), 51.9 (C-14), and 57.3 (C-17). HMBC correlations from the methylenic proton at δ_H_ 5.33 (H-7), with the carbons at δ_C_ 143.8 (C-8), 76.1 (C-9), and 51.9 (C-14), allowed the linking of rings B and C. Taking into account the molecular formula and the seven degrees of unsaturation previously established, as well as the presence of one unsaturation in the alkyl chain, the epoxy ring was suggested to be at the C-5/C-6 position for compound **1**. Furthermore, correlations from the methyl protons at δ_H_ 1.03 (CH_3_-21), with carbons at δ_C_ 57.3 (C-17), 41.8 (C-20), and 139.1 (C-22), and from the methyl protons at 0.89 (CH_3_-26 and 27), with carbons at δ_C_ 43.1 (C-24), and 29.8 (C-25), allowed determining the planar structure of **1** (Figure 2).

**Figure 3:**
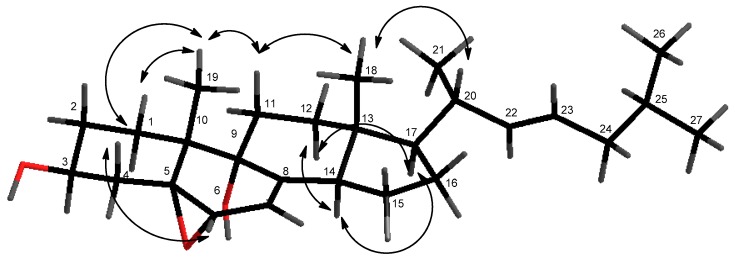
Key NOESY correlations of compound **1**.

The relative configuration of compound **1** was suggested by proton coupling constants and nuclear Overhauser effect spectroscopy (NOESY) spectrum analyses (Figure 3). Since the signal at δ_H_ 4.0 (H-3) appeared as a multiplet in the ^1^H NMR spectrum, its coupling constants could not be directly determined. However, the proton axial at δ_H_ 2.11 (CH_2_-4) showed two anti-coupling constants (*J* = 11.4 and 13.8 Hz). These data indicated an axial orientation for the proton H-3. In the NOESY spectrum, the signal at δ_H_ 2.11 (H-4_ax_) showed correlations with the proton at δ_H_ 3.65 (H-6) and the methyl at δ_H_ 1.12 (CH_3_-19), suggesting their co-facial dispositions. Furthermore, NOESY correlations of H-11_ax_ with both methyls at δ_H_ 0.66 (CH_3_-18) and at δ_H_ 1.12 (CH_3_-19) were observed. Therefore, the α-configuration was deduced for the hydroxyl group at C-9 and at the 5-6-epoxy function. The transfused junction for the C/D rings was established by NOESY correlations from the signal at δ_H_ 2.49 (H-14) with the proton at δ_H_ 1.61 (H-12_ax_), and between the signals at δ_H_ 1.91 (H-11_ax_) and δ_H_ 0.66 (CH_3_-18). Additionally, protons at δ_H_ 1.37 (H-17) and at δ_H_ 2.49 (H-14) were determined to be co-facial, as indicated by their correlation in the NOESY spectrum. In addition, NOESY correlations between CH_3_-18, CH_3_-19, and CH-20 indicated that they are oriented on the same side of the molecule. The 20*R* absolute configuration was supported by the chemical shift at δ_H_ 1.03 of CH_3_-21 [18,19,20]. Furthermore, a strong coupling constant between H-22/H-23 (*J* = 15.0 Hz) assigned the *E*-configuration for the C-22/C-23 double bond. Therefore, according to this intensive analysis of 2D NMR spectra, the structure of the new compound **1** was identified as 5α,6α-epoxycholesta-7,22(*E*)-dien-3β,9α-diol.

Compound **2** was isolated as white microcrystals. The HRESIMS of compound **2** exhibited a fragment ion peak at *m*/*z* 393.3070 [M-2H_2_O + H]^+^ (calculated 393.3157 for C_28_H_41_O), corresponding for compound **2** to a molecular formula of C_28_H_44_O_3_ with seven degrees of unsaturation. The ^1^H and ^13^C NMR data showed that compound **2** shares the same Δ^7^ sterol nucleus as **1**, with the presence of an epoxy group at C-5/C-6 and a hydroxyl group at C-9 (Table 1). However, significant differences were noted in the ^1^H NMR spectrum for signals of the side chain. In particular, the presence of gem olefinic protons at δ_H_ 4.67 and 4.73 was observed in the ^1^H NMR spectrum for compound **2**. The HMBC correlations from these olefinic protons with the carbons at δ_C_ 32.1 (C-23) and 34.9 (C-25) located the double bond at position C-24/C-28. Careful analysis of 2D NMR spectra revealed the structure of compound **2** as 5α,6α-epoxycholesta-7,24(28)-dien-3β,9α-diol (Figure 1).

Compound **3** was obtained as white microcrystals. The HRESIMS of **3** exhibited a fragment ion peak at *m*/*z* 427.3597 [M-H_2_O + H]^+^ (calculated 427.3576 for C_29_H_47_O_2_), corresponding for compound **3** to a molecular formula of C_29_H_48_O_3_ with six degrees of unsaturation. Examination of the NMR data indicated that compound **3** also shared the same Δ^7^-5,6-epoxyhydroxy sterol nucleus as compounds **1** and **2**, but possesses a different side chain (Figure 1). Comparison of the NMR data of compound **3** with that of **2** revealed the presence of a methine and an ethyl group in **3** instead of the C-24/C-28 double bond resonance in the structure of **2**. The ethyl group (CH_2_-28-CH_3_-29) was determined to be located at C-24 from the COSY correlations between the methyl at δ_H_ 0.88 (CH_3_-29) and the methylene at δ_H_ 1.35–1.18 (CH_2_-28), which in turn correlated with the methine at δ_H_ 0.96 (CH-24). Although only one diastereomer was isolated, the difference between C-26 and C-27 chemical shifts, which is 0.70 ppm, suggests the 24 *R* configuration for compound **3** (instead of 0.55 ppm in the case of *S*-configuration) [21,22]. The new sterol **3** was thus deduced to be (24*R*)-5α,6α-epoxy-24-ethyl-cholesta-7-en-3β,9α-diol (Figure 1).

**Figure 4:**
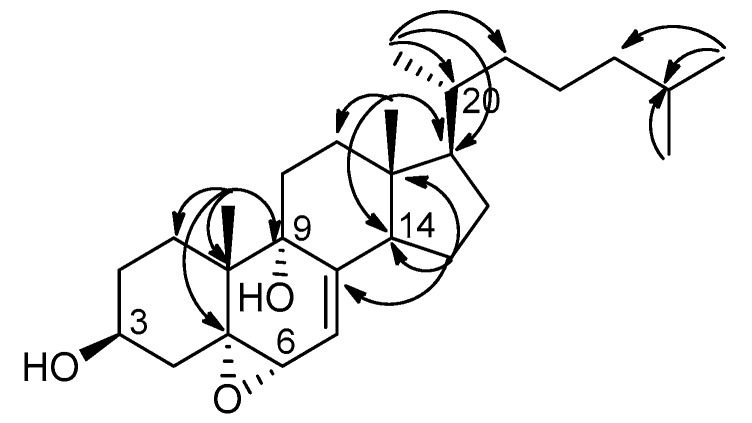
Selected HMBC correlations in compound **4**.

Compound **4** was isolated as white microcrystals. The HRESIMS of compound **4** exhibited a fragment ion peak at *m*/*z* 399.3259 [M-H_2_O + H]^+^ (calculated 399.3263 for C_27_H_43_O_2_), corresponding for compound **4** to a molecular formula of C_27_H_44_O_3_ with six degrees of unsaturation. The ^1^H NMR signals of **4** were close to those of **1**, except for the presence of two methylenes instead of the C-22/C-23 double bond. Analysis of 2D NMR spectra, in particular of HMBC correlations (Figure 4), established the structure of compound **4** as 5α,6α-epoxycholesta-7-en-3β,9α-diol (Figure 1). Compound **4** was previously prepared from the free radical chain oxidation process of 7-dehydrocholesterol, but this is its first report as a natural product [23].

Compound **5** was isolated as white microcrystals. The HRESIMS of compound **5** exhibited a fragment ion peak at *m*/*z* 411.3284 [M-H_2_O + H]^+^ (calculated 411.3263 for C_28_H_43_O_2_), corresponding for compound **5** to a molecular formula of C_28_H_44_O_3_ with seven degrees of unsaturation. A comparison of the ^1^H and ^13^C NMR data of compound **5** with that of sterol **1** revealed the presence of an additional methyl group at δ_H_ 0.94 (d, *J* = 7.2 Hz) and its corresponding resonance at δ_C_ 18.6, suggesting the replacement of a methylene at C-24 in compound **1** by a methine linked to a methyl group for compound **5**. The location of the additional methyl group in C-24 was confirmed by HMBC correlations from the methyl at δ_H_ 0.94 (CH_3_-28), with the carbons at δ_C_ 133.4 (C-23) and 34.5 (C-25). The configuration at C-24 of compound **5** was determined by comparison of the ^13^C NMR chemical shifts with those of the related structures [21,22]. Accordingly, due to the chemical difference shift of 0.6 ppm between the C-26 and C-27 carbon atoms and the chemical shift of C-28 at δ_C_ 18.6 ppm, the *S*-configuration at C-24 was suggested for compound **5**. The new sterol **5** was thus deduced to be (24*S*)-5α,6α-epoxyergosta-7,22-dien-3β,9α-diol (Figure 1).

Compound **6** was isolated as white microcrystals. The HRESIMS of compound **6** exhibited a fragment ion peak at *m/z* 413.3414 [M-H_2_O + H]^+^ (calculated 413.3420 for C_28_H_45_O_2_), corresponding to a molecular formula of C_28_H_46_O_3_ with six degrees of unsaturation. A detailed examination of ^1^H and ^13^C NMR data (Table 1 and Table 2) showed that compound **6** also belongs to the 9α-hydroxy-5α,6α-epoxysterols. Comparison with compound **4** revealed the presence of an additional methyl group in C-24. The configuration of C-24 in compound **6** was determined to be *R*, since the chemical shift difference between C-26 and C-27 carbon atoms is 2.0 ppm, whereas in the *S* configuration the expected value for a saturated 24-methyl side chain should be 3.0 ppm [21,22]. The new sterol **6** was thus deduced to be (24*R*)-5α,6α-epoxy-24-methyl-cholesta-7-en-3β,9α-diol (**6**).

Three known compounds 5α,6α-epoxystigmasta-7-en-3β-ol (**7**), 5α,6α-epoxystigmasta-7,22-dien-3β-ol (**8**), and 5α,6α-epoxyergosta-7-en-3β-ol (**9**) were also isolated and characterized from the MeOH extract of *I. echinata*. Their structures were determined by spectral data and comparison with those reported in the literature from the marine sponge *Ircinia aruensis* [12].

Evaluation of all steroids isolated from *I. echinata* was performed against three cancer cell lines: MCF-7 (human breast cancer cells), HepG-2 (human liver hepatocellular carcinoma cells), and Lu-1 (human lung adenocarcinoma cells). All compounds were inactive until 32 µg.mL^−1^, except the two new steroids (24*R*)-5α,6α-epoxy-24-ethyl-cholesta-7-en-3β,9α-diol (**3**) and 5α,6α-epoxycholesta-7-en-3β,9α-diol (**4**) (Table 3). Compound **3** exhibited a selective inhibition towards the MCF-7 cells with an IC_50_ value of 15.88 ± 1.36 µg.mL^−1^, whereas the steroid **4** showed a similar range of cytotoxic activity against the three cancer cell lines (Table 3 and Figure S43). Comparison of the active sterol **3** with the inactive non-hydroxylated sterol **7** could show, in this case, the importance of hydroxylation in position C-9. Furthermore, an additional ethyl group on the alkyl chain could exert a selective activity against MCF-7.

## 3. Materials and Methods

### 3.1. General Experimental Procedures

Flash chromatography was carried out on a Buchi C-615 pump system (Rungis, France). Analytical and semi-preparative reverse-phase (Luna C18 or biphenyl Kinetex, Phenomenex, Le Pecq, France) columns were performed with an Alliance HPLC apparatus (model 2695, Waters, Saint-Quentin-en-Yvelines, France), equipped with a photodiode array detector (model 2998, Waters), an evaporative light-scattering detector (model Sedex 80, Sedere, Alfortville, France), and the Empower software. Mass spectra were recorded on an API Q-STAR PULSAR I (Applied Biosystem, Concord, ON, Canada) and on a Maxis II-ETD (Bruker, Wissenbourg, France). 1D and 2D NMR (COSY, HSQC, HMBC, NOESY) spectra were recorded on a Bruker AVANCE 600 (Bruker, Wissenbourg, France).

### 3.2. Biological Materials

Specimens of *I. echinata* (Keller, 1889) (Order Dictyoceratida, Family Irciniidae) were collected at Bai Tu Long (Quang Ninh, Vietnam) in August 2014. The sponge sample was identified by Professor Do Cong Thung of the Institute of Marine Environment and Resources, and a voucher specimen (HM01-39α) has been deposited at the Institute of Marine Biochemistry (VAST, Vietnam).

### 3.3. Isolation of 5α,6α-Epoxysterols from the Vietnamese Marine Sponge *Ircinia echinata*

The air-dried sponge of *I. echinata* (0.5 kg) was extracted with MeOH at room temperature (3 times × 2 L). The solvent was removed under reduced pressure to give 15 g of the MeOH crude extract. An aliquot of 5 g was subjected to flash chromatography on silica gel (from 0% to 100% MeOH in CH_2_Cl_2_) to yield 12 fractions. Fraction F10 (150 mg) and fraction F12 (380 mg) were each chromatographed on a silica gel column (n-hexane/acetone gradient), and then on a Sephadex LH-20 column, using MeOH/CH_2_Cl_2_ (9/1) as eluent to afford subfraction F10.2.2 (9 mg) and F12.2.1 (9 mg), respectively. Subfraction F10.2.2 was purified by a semi-preparative reverse phase HPLC (Luna 5 µ C18 Phenomenex, 250 × 10 mm, flow rate 3 mL.min^−1^) eluting with a gradient solvent system MeOH/H_2_O/HCOOH from 92/8/0.1 to 95/5/0.1 for 20 min to give compounds **7** (0.8 mg), **8** (0.5 mg), and **9** (0.8 mg). Similarly, subfraction F12.2.1 was then purified by HPLC on a semi-preparative reverse phase column, using the gradient solvent system MeOH/H_2_O/HCOOH from 85/15/0.1 to 95/5/0.1 for 20 min to yield the new pure compounds **1** (2.0 mg), **2** (1.5 mg), and **3** (2.0 mg), as well as impure sub-fractions A and B. Sub-fraction A was purified using reverse phase HPLC (Kinetex 5 µ biphenyl Phenomenex, 250 × 4.6 mm, flow rate 1 mL.min^−1^) with MeOH/H_2_O (75/25) as eluent, to furnished the new compounds **4** (0.8 mg) and **5** (0.5 mg). Sub-fraction B was purified using reverse phase HPLC (Kinetex 5 µ biphenyl Phenomenex, 250 × 4.6 mm, flow rate 1 mL.min^−1^) with MeOH/H_2_O (80/20) as eluent, to yield the new compound **6** (0.4 mg).

#### 3.3.1. 5α,6α-Epoxycholesta-7,22(*E*)-dien-3β,9α-diol (**1**)

Compound **1**: white microcrystals; m.p. 150–151 °C; [α]^25^_D_ −15.0 (*c* 0.1, MeOH). The HRESIMS results showed [M-2H_2_O + H]^+^ found at *m*/*z* 379.2953 (calculated 379.3001 for C_27_H_39_O), and [M-3H_2_O + H]^+^ found at *m*/*z* 361.2913 (calculated 361.2895 for C_27_H_37_). For ^1^H and ^13^C NMR, see Table 1 and Table 2.

#### 3.3.2. 5α,6α-Epoxycholesta-7,24(28)-dien-3β,9α-diol (**2**)

Compound **2**: white microcrystals; m.p. 181–182 °C; [α]^25^_D_ −11.0 (*c* 0.1, MeOH). The HRESIMS results showed [M-2H_2_O + H]^+^ found at *m*/*z* 393.3070 (calculated 393.3157 for C_28_H_41_O), and [M-3H_2_O + H]^+^ found at *m*/*z* 375.3035 (calculated 375.3052 for C_28_H_39_). For ^1^H and ^13^C NMR, see Table 1 and Table 2.

#### 3.3.3. (24*R*)-5α,6α-Epoxy-24-ethyl-cholesta-7-en-3β,9α-diol (**3**)

Compound **3**: white microcrystals; m.p. 172–173 °C; [α]^25^_D_ −17.0 (*c* 0.08, MeOH). The HRESIMS results showed [M-H_2_O + H]^+^ found at *m*/*z* 427.3597 (calculated 427.3576 for C_29_H_47_O_2_), and [M-2H_2_O + H]^+^ found at *m*/*z* 409.3499 (calculated 409.3470 for C_29_H_45_O), and [M-3H_2_O + H]^+^ found at *m*/*z* 391.3371 (calculated 391.3365 for C_29_H_43_). For ^1^H and ^13^C NMR, see Table 1 and Table 2.

#### 3.3.4. 5α,6α-Epoxycholesta-7-en-3β,9α-diol (**4**)

Compound **4**: white microcrystals; m.p. 158–159 °C; [α]^25^_D_ −46.0 (*c* 0.08, MeOH). The HRESIMS results showed [M-H_2_O + H]^+^ found at *m*/*z* 399.3259 (calculated 399.3263 for C_27_H_43_O_2_), [M-2H_2_O + H]^+^ found at *m*/*z* 381.3161 (calculated 381.3157 for C_27_H_41_O), and [M-3H_2_O + H]^+^ found at *m*/*z* 363.3053 (calculated 363.3052 for C_27_H_39_). For ^1^H and ^13^C NMR, see Table 1 and Table 2.

#### 3.3.5. (24*S*)-5α,6α-Epoxyergosta-7,22-dien-3β,9α-diol (**5**)

Compound **5**: white microcrystals; m.p. 190–192 °C; [α]^25^_D_ −32.0 (*c* 0.08, MeOH). The HRESIMS results showed [M-H_2_O + H]^+^ found at *m*/*z* 411.3284 (calculated 411.3263 for C_28_H_43_O_2_), [M-2H_2_O + H]^+^ found at *m*/*z* 393.3128 (calculated 393.3157 for C_28_H_41_O), and [M-3H_2_O + H]^+^ found at *m*/*z* 375.3044 (calculated 375.3052 for C_28_H_39_). For ^1^H and ^13^C NMR, see Table 1 and Table 2.

#### 3.3.6. (24*R*)-5α,6α-Epoxy-24-methyl-cholesta-7-en-3β,9α-diol (**6**)

Compound **6**: white microcrystals; m.p. 207–208 °C; [α]^25^_D_ −15.0 (*c* 0.05, MeOH). The HRESIMS showed [M-H_2_O + H]^+^ found at *m*/*z* 413.3414 (calculated 413.3420 for C_28_H_45_O_2_), [M-2H_2_O + H]^+^ found at *m*/*z* 395.3306 (calculated 395.3314 for C_28_H_43_O), and [M-3H_2_O + H]^+^ found at *m*/*z* 377.3202 (calculated 377.3208 for C_28_H_41_). For ^1^H and ^13^C NMR, see Table 1 and Table 2.

### 3.4. Evaluation of Cytotoxic Activity

Cytotoxicity assays were carried out in triplicate in a 96-well microtiter plates against HepG-2, Lu-1, and MCF-7, using a modification of the published method [24]. Cells were maintained in Dulbecco′s D-MEM medium, supplemented with 10% fetal calf serum, L-glutamine (2 mM), penicillin G (100 UI.mL^−1^), streptomycin (100 μg.mL^−1^), and gentamicin (10 μg.mL^−1^). Stock solutions of the compounds were prepared in DMSO/H_2_O (1/9), and the cytotoxicity assays were carried out against cancer cells (3 × 10^3^ cells.mL^−1^). After 72 h of incubation at 37 °C in air/CO_2_ (95:5), with or without the test compounds, cell growth was estimated by colorimetric measurement of stained living cells using neutral red. Optical density was determined at 540 nm with a Titertek Multiscan photometer. The IC_50_ value was defined as the concentration of the sample necessary to inhibit cell growth to 50% of the control. Ellipticine was used as a reference compound.

## Figures and Tables

**Table 1:** ^1^H NMR data of compounds **1**–**6** recorded in CD_3_OD (δ_H_, multiplicity, *J* in Hz).

N^o^	1	2	3	4	5	6
1	2.20 dt (4.3, 13.5)	2.20 dt (4.3, 13.5)	2.20 dt (4.3, 13.8)	2.20 dt (4.2, 13.8)	2.20 dt (4.2, 13.8)	2.20 dt (4.2, 13.8)
	1.35 m	1.35 m	1.36 m	1.35 m	1.36 m	1.36 m
2	1.86 m	1.86 m	1.88 m	1.85 m	1.86 m	1.86 m
	1.51 m	1.50 m	1.52 m	1.52 m	1.51 m	1.51 m
3	4.00 m	4.00 m	4.00 m	4.00 m	4.0 m	4.0 m
4	1.66 m	1.66 m	1.66 m	1.67 m	1.66 m	1.67 m
	2.11 dd (11.4, 13.8)	2.12 dd (11.2, 13.5)	2.12 dd (11.2, 13.8)	2.12 dd (11.4, 13.2)	2.12 dd (11.4, 13.2)	2.12 dd (11.4, 13.8)
6	3.65 m	3.64 m	3.66 m	3.66 m	3.65 m	3.65 m
7	5.33 m	5.33 m	5.34 dd (1.8, 4.8)	5.34 dd (2.4, 4.8)	5.34 dd (2.4, 4.8)	5.33 dd (1.8, 4.8)
11	1.60 m	1.97 m	1.97 m	1.97 m	1.60 m	1.60 m
	1.91 m	1.59 m	1.60 m	1.60 m	1.90 m	1.97 m
12	1.61 m	1.60 m	1.59 m	1.59 m	1.61 m	1.60 m
	1.83 m	1.86 m	1.86 m	1.86 m	1.83 m	1.86 m
14	2.49 m	2.50 m	2.49 m	2.50 m	2.50 m	2.49 m
15	1.55 m	1.61 m	1.61 m	1.61 m	1.56 m	1.61 m
	1.48 m	1.51 m	1.53 m	1.53 m	1.48 m	1.52 m
16	1.34 m	1.36 m	1.35 m	1.34 m	1.34 m	1.34 m
	1.80 m	1.90 m	1.91 m	1.90 m	1.80 m	1.91 m
17	1.37 m	1.36 m	1.36 m	1.34 m	1.37 m	1.34 m
18	0.66 s	0.65 s	0.65 s	0.65 s	0.66 s	0.65 s
19	1.12 s	1.12 s	1.12 s	1.12 s	1.12 s	1.12 s
20	2.05 m	1.45 m	1.41 m	1.41 m	2.04 m	1.41 m
21	1.03 d (6.5)	0.99 d (6.8)	0.97 d (6.5)	0.96 d (6.6)	1.03 d (6.6)	0.96 d (6.6)
22	5.24 dd (8.0, 15.0)	1.18 m, 1.60 m	1.60 m, 1.07 m	1.41 m, 1.14 m	5.20 m	1.11 m, 1.40 m
23	5.32 m	1.92 m, 2.13 m	1.25 m	1.40 m, 1.19 m	5.21 m	1.28 m, 1.53 m
24	1.84 m		0.96 m	1.16 m	1.85 m	1.23 m
25	1.58 m	2.24 m	1.71 m	1.53 m	1.47 m	1.55 m
26	0.89 d (6.7)	1.03 d (6.7)	0.86 d (6.8)	0.89 d (6.6)	0.88 d (7.2)	0.88 d (7.2)
27	0.89 d (6.7)	1.04 d (6.7)	0.84 d (6.8)	0.89 d (6.6)	0.86 d (7.2)	0.83 d (7.2)
28		4.67 brs, 4.73 brs	1.35 m, 1.18 m		0.94 d (7.2)	0.81 d (7.2)
29			0.88 m			

**Table 2:** ^13^C NMR data for compounds **1**–**6** recorded in CD_3_OD.

N^o^	1	2	3	4	5	6
1	28.2 t	28.2 t	28.3 t	28.2 t	28.3 t	28.3 t
2	31.6 t	31.6 t	31.6 t	31.6 t	31.6 t	31.6 t
3	68.2 d	68.2 d	68.2 d	68.2 d	68.2 d	68.2 d
4	40.7 t	40.7 t	40.8 t	40.7 t	40.7 t	40.7 t
5	78.9 s	78.9 s	78.9 s	78.9 s	78.9 s	78.9 s
6	73.7 d	73.7 d	73.7 d	73.7 d	73.7 d	73.7 d
7	121.0 d	121.1 d	121.0 d	121.0 d	121.0 d	121.0 d
8	143.8 s	143.8 s	143.8 s	143.8 s	143.8 s	143.8 s
9	76.1 s	76.0 s	76.1 s	76.0 s	76.1 s	76.1 s
10	41.4 s	41.4 s	41.4 s	41.4 s	41.4 s	41.4 s
11	29.1 t	28.9 t	29.0 t	28.9 t	29.2 t	29.0 t
12	36.5 t	36.6 t	36.6 t	36.6 t	36.5 t	36.5 t
13	44.8 s	44.9 s	44.9 s	44.9 s	44.8 s	44.8 s
14	51.9 d	51.8 d	51.8 d	51.8 d	51.9 d	51.8 d
15	24.0 t	24.1 t	24.1 t	24.1 t	24.0 t	24.0 t
16	29.3 t	29.1 t	29.2 t	29.1 t	29.6 t	29.1 t
17	57.3 d	57.4 d	57.4 d	57.5 d	57.4 d	57.5 d
18	12.2 q	12.0 q	12.2 q	12.0 q	12.2 q	12.0 q
19	22.2 q	22.2 q	22.2 q	22.2 q	22.2 q	22.2 q
20	41.8 d	37.3 d	37.9 d	37.5 d	41.9 d	37.5 d
21	21.5 q	19.3 q	19.4 q	19.3 q	21.5 q	19.3 q
22	139.1 d	35.8 t	35.0 t	37.2 t	137.2 d	35.0 t
23	127.8 d	32.1 t	27.2 t	24.9 t	133.4 d	31.4 t
24	43.1 t	157.3 s	47.4 d	40.6 t	44.6 d	40.3 d
25	29.8 d	34.9 d	30.2 d	29.2 d	34.5 d	33.7 d
26	22.7 q	22.3 q	20.1 q	22.9 q	20.7 q	20.5 q
27	22.7 q	22.4 q	19.4 q	23.2 q	20.1 q	18.5 q
28		106.1 t	24.1 t		18.6 q	15.8 q
29			12.7 q			

**Table 3:** Cytotoxic evaluation of the active 5α,6α-epoxysterols **3**–**4** against human cancer cell lines (IC_50_ values are expressed in µg.mL^−1^).

Compound	Human Cancer Cell Lines		
	MCF-7	HepG-2	Lu-1
**3**	15.88 ± 1.36	>32	>32
**4**	15.88 ± 0.09	15.95 ± 0.20	22.92 ± 0.09
Ellipticine	0.34 ± 0.01	0.38 ± 0.05	0.41 ± 0.04

Ellipticine was used as positive control. Values presented as the mean ± (SEM) (*n* = 3).

## Supplementary Materials

The following are available online at http://www.mdpi.com/1660-3397/16/11/424/s1, HRESIMS as well as 1D and 2D NMR spectra of compounds **1**–**6** along with growth inhibition curves of active compounds **3** and **4** against three human cancer cell lines.

## References

[1] Kawakami A., Miyamoto T., Higuchi R., Uchiumi T., Kuwano M., Soet R.W.M.V. (2001). Structure of a novel multidrug resistance modulator, irciniasulfonic acid, isolated from a marine sponge *Ircinia* sp.. Tetrahedron Lett..

[2] Sica D., Piccialli V., Pronzato R. (1987). Sterols from the sponges *Ircinia pipetta* & *Dysidea avara*. Identification of cholestatrienol. Comp. Biochem. Physiol..

[3] Venkateswarlu Y., Reddy M.V.R., Rao M.N. (1996). A new epoxy sterol from the sponge *Ircinia fasciculata*. J. Nat. Prod..

[4] Hahn D., Chin J., Kim H., Yang I., Won D.H., Ekins M., Choi H., Nam S.J., Kang H. (2007). Sesquiterpenoids with PPARδ agonistic effect from a Korean marine sponge *Ircinia* sp.. Tetrahedron Lett..

[5] Buchanan M.S., Edser A., King G., Whitmore J., Quin R.J. (2001). Cheilanthane sesterterpenes, protein kinase inhibitors, from a marine sponge of the genus *Ircinia*. J. Nat. Prod..

[6] Lai Y.-Y., Lu M.-C., Wang L.-H., Chen J.-J., Fang L.-S., Wu Y.-C., Sung P.-J. (2015). New scalarane sesterterpenoids from the Formosan sponge *Ircinia felix*. Mar. Drugs.

[7] Issa H.H., Tanaka J., Higa T. (2003). New cytotoxic furanosesterterpenes from an Okinawan marine sponge *Ircinia* sp.. J. Nat. Prod..

[8] Feng Y., Caroll A.R., Pass D.M., Archbold J.K., Avery V.M., Quin R.J.J. (2008). Polydiscamides B−D from a marine sponge *Ircinia* sp. as potent human sensory neuron-specific G protein coupled receptor agonists. J. Nat. Prod..

[9] Rashid M.A., Gustafson K.R., Boyd M.R. (2001). New chondropsin macrolide lactams from marine sponges in the genus *Ircinia*. Tetrahedron Lett..

[10] Chevallier C., Bugni T.S., Feng X., Harper M.K., Orendt A.M., Ireland C.M. (2006). Tedanolide C:  A potent new 18-membered-ring cytotoxic macrolide isolated from the Papua New Guinea marine sponge *Ircinia* sp.. J. Org. Chem..

[11] Marquez F.D.M., Martinez M.A. (2007). Antileishmanial epidioxysterols from the Colombian marine sponge *Ircinia campana* are oxidation products from naturally occurring Δ5,7 sterols. Vitae.

[12] Xu S., Liao X., Du B., Zhou X., Huang Q., Wu C. (2008). A series of new 5,6-epoxysterols from a Chinese sponge *Ircinia aruensis*. Steroids.

[13] Kobayashi J., Shinonaga H., Shigemori H., Umeyama A., Shoji N., Arihara S. (1995). Xestobergsterol C, a new pentacyclic steroid from the Okinawan marine sponge *Ircinia* sp. and absolute stereochemistry of Xestobergsterol A. J. Nat. Prod..

[14] Yang I., Choi H., Won D.H., Nam S.-J., Kang H. (2014). An antibacterial 9,11-secosterol from a marine sponge *Ircinia* sp.. Bull. Korean Chem. Soc..

[15] Yang I., Choi H., Nam S.-J., Kang H. (2015). A new 9,11-secosterol with a 1,4-quinone from a Korean marine sponge *Ircinia* sp.. Arch. Pharm. Res..

[16] Do T.T., Duong T.D., Do C.T., Pham H.Y., Nguyen X.N., Dan T.T.H., Bui H.T., Hoang L.T.A., Nguyen T.C., Chau V.M. (2016). Sterols from the Vietnamese sponge *Ircinia echinata*. Vietnam J. Chem..

[17] Phan V.K., Duong T.D., Do T.T., Tran H.Q., Nguyen T.T.N., Tran M.H., Hoang L.T.A., Pham H.Y., Do T.T., Nguyen X.N. (2017). Constituents from *Ircinia echinata* and their antiproliferative effect on six human cancer cell strains. Lett. Org. Chem..

[18] Vanderah D.J., Djerassi C. (1978). Marine natural products. Synthesis of four naturaly occurring 20β-H Cholanic acid derivatives. J. Org. Chem..

[19] Aiello A., Fattorusso E., Menna M. (1992). Four new bioactive polyhydroxylated sterols from the black coral *Antipathes subpinnata*. J. Nat. Prod..

[20] Mansoor T.A., Lee Y.M., Hong J., Lee C.-O., Im K.S., Jung J.H. (2006). 5,6:8,9-Diepoxy and other cytotoxic sterol from the marine sponge *Homaxinella* sp.. J. Nat. Prod..

[21] Wright J.L.C., McInnes A.G., Shimizu S., Smith D.G., Walter J.A. (1978). Identification of C-24 alkyl epimers of marine sterol by 13C nuclear magnetic resonance spectroscopy. Can. J. Chem..

[22] Ioannou E., Abdel-Razik A.F., Zervou M., Christofidis D., Alexi X., Vagias C., Alexis M.N., Roussis V. (2009). 5α,8α-epidioxysterols from the gorgonian *Eunicella cavolini* and the ascidian *Trididemnum inarmatum*: Isolation and evaluation of their antiproliferative activity. Steroids.

[23] Xu L., Korade Z., Porter N.A. (2010). Oxysterols from free radical chain oxidation of 7-dehydrocholesterol: Product and mechanistic studies. J. Am. Chem. Soc..

[24] Mosmann T. (1983). Rapid colorimetric assay for cellular growth and survival: Application to proliferation and cytotoxicity assays. J. Immunol. Methods.

