# Supplementary material for: New 9α-Hydroxy-5α,6α-epoxysterols from the Vietnamese Marine Sponge Ircinia echinata
Source: Mar Drugs. 2018 Nov 1;16(11):424. doi: 10.3390/md16110424 (PMC6267468; doi:10.3390/md16110424)
Supplement: Supplementary file 1 [file marinedrugs-16-00424-s001.pdf]

marine drugs

Supplementary Material

New 9α-Hydroxy-5α,6α-epoxyhydroxysterols from the Vietnamese Marine Sponge *Ircinia echinata*

Thi Thanh Van Trinh [1,†], Bich Ngan Truong [1,†], Arlette Longeon [2], Thi Mai Huong Doan [1], Alexandre Deville [2], Van Minh Chau [1], Van Cuong Pham [1,*] and Marie-Lise Bourguet-Kondracki [2,*]

[1] Advanced Center for Bioorganic Chemistry of the Institute of Marine Biochemistry, Vietnam Academy of Science and Technology, 18 Hoang Quoc Viet, Caugiay 8424, Hanoi, Vietnam; trtvan76@yahoo.com (T.T.V.T.); tbngan1977@yahoo.com (B.N.T.); doanhuong7@yahoo.com (T.M.H.D.); cvminh@vast.vn (V.M.C.)

[2] Molécules de Communication et Adaptation des Microorganismes, UMR 7245 CNRS, Muséum National d'Histoire Naturelle, 57 rue Cuvier (CP54), 75005 Paris, France; arlette.longeon@mnhn.fr (A.L.); alexandre.deville@mnhn.fr (A.D.)

* Correspondence: phamvc@imbc.vast.vn (V.C.P.); bourguet@mnhn.fr (M.-L.B.-K.); Tel.: +84-4-37917049 (V.C.P.); +33-1-40-79-56-06 (M.-L.B.-K); Fax: +84-4-37917054 (V.C.P.); 33-1-40-79-31-35 (M.-L.B.-K.)

† These authors contributed equally to this paper.

Content:

Figure S1. ^{1}H NMR spectrum (600 MHz) of 5α,6α-Epoxycholesta-7,22(*E*)-dien-3β,9α-diol (**1**) in CD_3OD.

Figure S2. DEPT spectrum (150.9 MHz) of **1** in CD_3OD.

Figure S3. COSY spectrum (600 MHz) of **1** in CD_3OD.

Figure S4. HSQC spectrum (600 MHz) of **1** in CD_3OD.

Figure S5. HMBC spectrum (600 MHz) of **1** in CD_3OD.

Figure S6. NOESY spectrum (600 MHz) of **1** in CD_3OD.

Figure S7. HR-ESI mass spectrum of **1**.

Figure S8. ^{1}H NMR spectrum (600 MHz) of 5α,6α-Epoxycholesta-7,24(28)-dien-3β,9α-diol (**2**) in CD_3OD.

Figure S9. DEPT spectrum (150.9 MHz) of **2** in CD_3OD

Figure S10. COSY spectrum (600 MHz) of **2** in CD_3OD.

Figure S11. HSQC spectrum (600 MHz) of **2** in CD_3OD.

Figure S12. HMBC spectrum (600 MHz) of **2** in CD_3OD.

Figure S13. NOESY spectrum (600 MHz) of **2** in CD_3OD.

Figure S14. HR-ESI mass spectrum of **2**.

Figure S15. ^{1}H NMR spectrum (600 MHz) of (24*R*)-5α,6α-Epoxy-24-ethyl-cholesta-7-en-3β,9α-diol (**3**) in CD_3OD.

Figure S16. DEPT spectrum (150.9 MHz) of **3** in CD_3OD.

Figure S17. COSY spectrum (600 MHz) of **3** in CD_3OD.

Figure S18. HSQC spectrum (600 MHz) of **3** in CD_3OD.

Figure S19. HMBC spectrum (600 MHz) of **3** in CD_3OD.

Figure S20. NOESY spectrum (600 MHz) of **3** in CD_3OD.

Figure S21. HR-ESI mass spectrum of **3**.

Figure S22. ^{1}H NMR spectrum (600 MHz) of 5α,6α -Epoxycholesta-7-en-3β,9α-diol (**4**) in CD_3OD.

Figure S23. DEPT spectrum (150.9 MHz) of **4** in CD_3OD.

Figure S24. COSY spectrum (600 MHz) of **4** in CD_3OD.

Figure S25. HSQC spectrum (600 MHz) of **4** in CD_3OD.

Figure S26. HMBC spectrum (600 MHz) of **4** in CD_3OD.

Figure S27. NOESY spectrum (600 MHz) of **4** in CD_3OD.

Figure S28. HR-ESI mass spectrum of **4**.

Figure S29. ^{1}H NMR spectrum (600 MHz) of (24*S*)-5α,6α-Epoxyergosta-7,22-dien-3β,9α-diol (**5**) in CD_3OD.

Figure S30. DEPT spectrum (150.9 MHz) of **5** in CD_3OD.

Figure S31. COSY spectrum (600 MHz) of **5** in CD_3OD.

Figure S32. HSQC spectrum (600 MHz) of **5** in CD_3OD.

Figure S33. HMBC spectrum (600 MHz) of **5** in CD_3OD.

Figure S34. NOESY spectrum (600 MHz) of **5** in CD_3OD.

Figure S35. HR-ESI mass spectrum of **5**.

Figure S36. ^{1}H NMR spectrum (600 MHz) of (24*R*)-5α,6α-Epoxy-24-methyl-cholesta-7-en-3β,9α-diol (**6**) in CD_3OD.

Figure S37. DEPT spectrum (150.9 MHz) of **6** in CD_3OD.

Figure S38. COSY spectrum (600 MHz) of **6** in CD_3OD.

Figure S39. HSQC spectrum (600 MHz) of **6** in CD_3OD.

Figure S40. HMBC spectrum (600 MHz) of **6** in CD_3OD.

Figure S41. NOESY spectrum (600 MHz) of **6** in CD_3OD.

Figure S42. HR-ESI mass spectrum of **6**.

Figure S43. Growth inhibition ~~of~~ curves of the active compounds **3** and **4** against three human cancer cell lines.

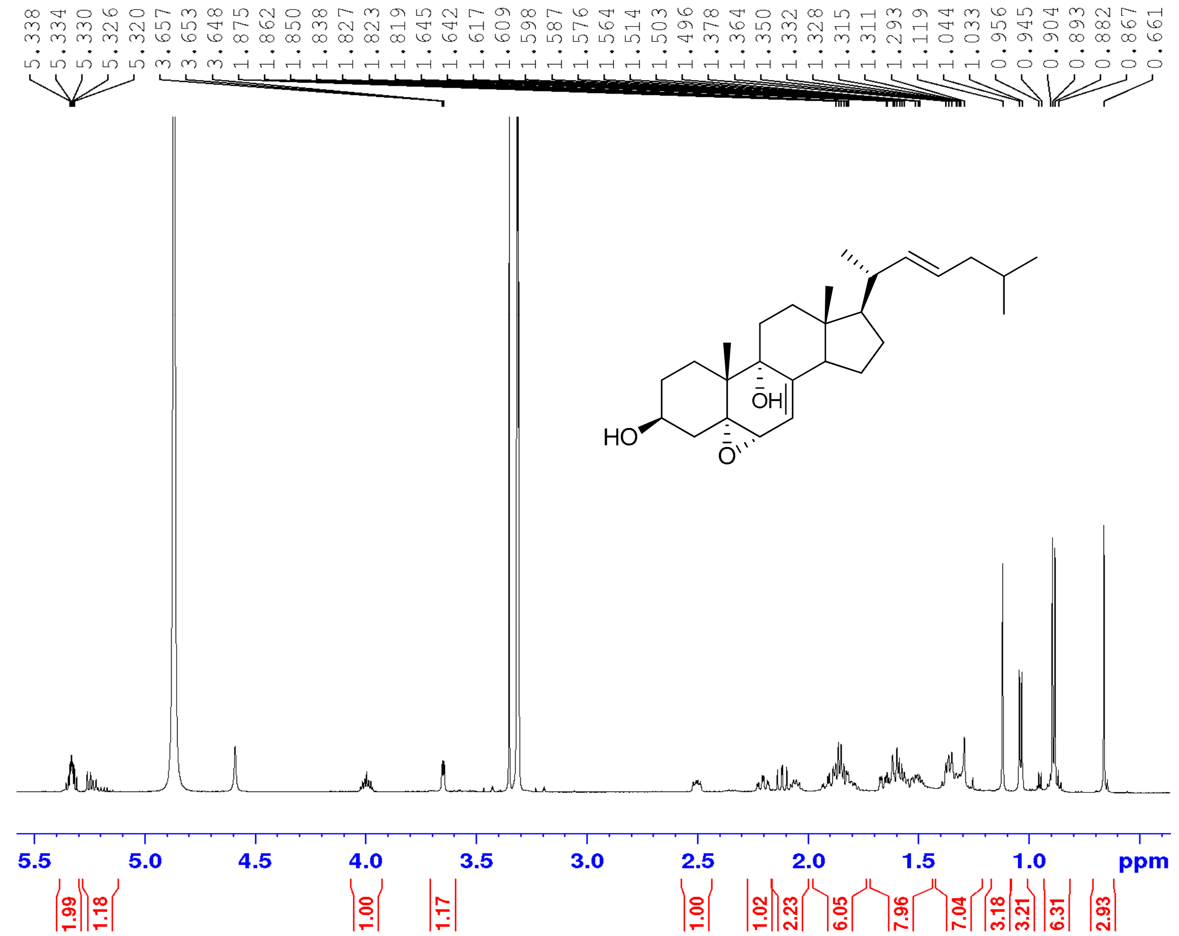

Figure S1. ^{1}H NMR spectrum (600 MHz) of 5*α*,6*α*-Epoxycholesta-7,22(*E*)-dien-3β,9*α*-diol (**1**). in CD3OD.

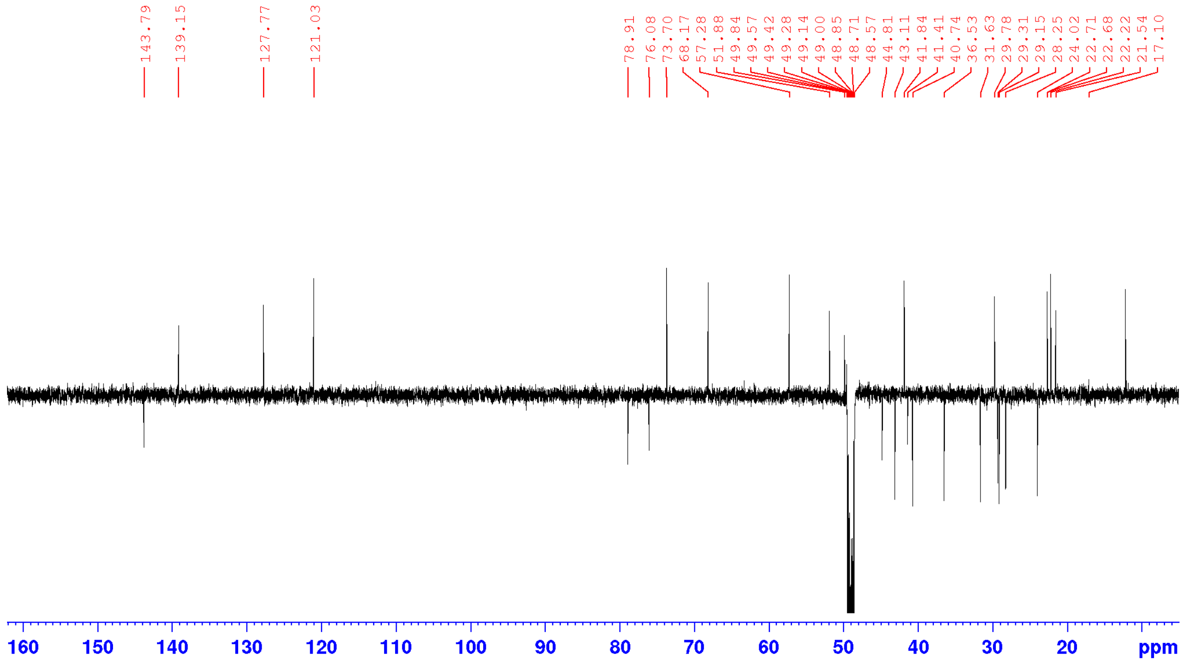

Figure S2. DEPT spectrum (150.9 MHz) of **1** in CD3OD.

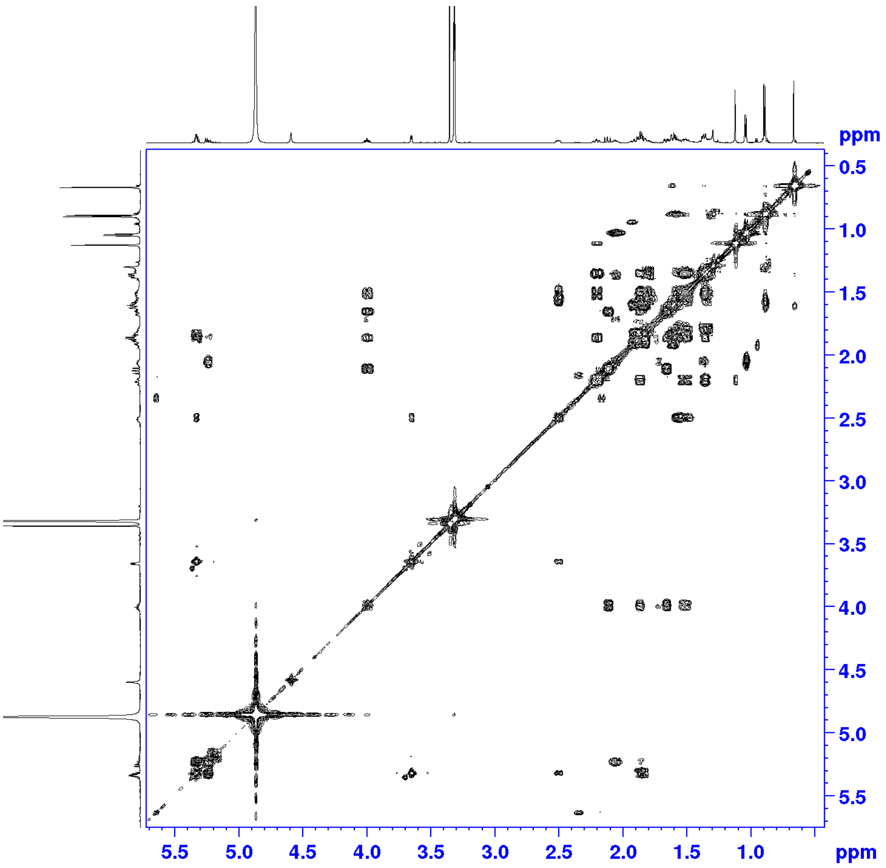

Figure S3. COSY spectrum (600 MHz) of **1** in CD_3OD.

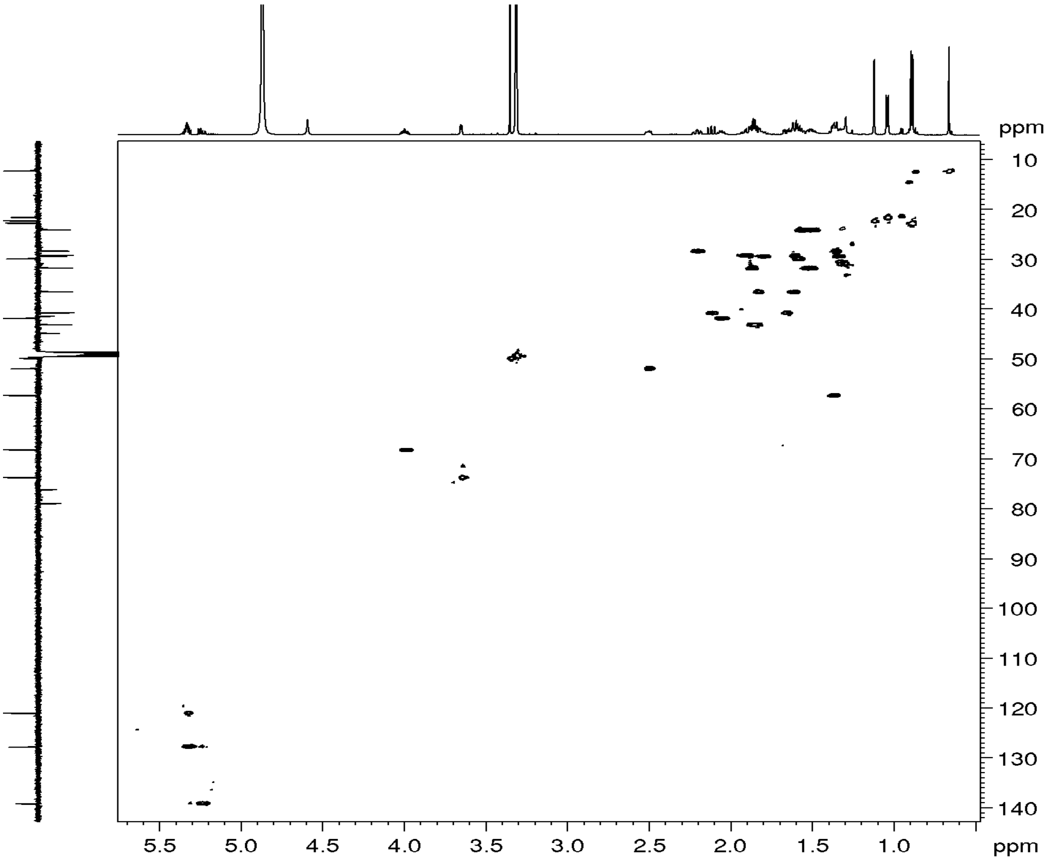

Figure S4. HSQC spectrum (600 MHz) of **1** in CD_3OD.

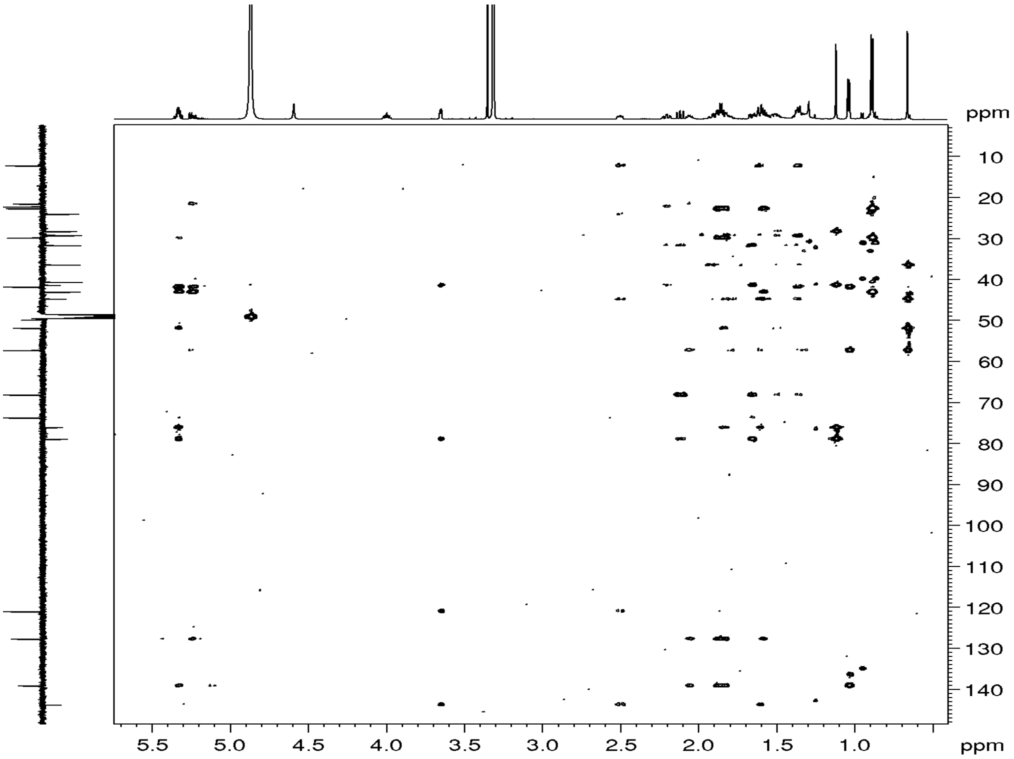

Figure S5. HMBC spectrum (600 MHz) of **1** in CD_3OD.

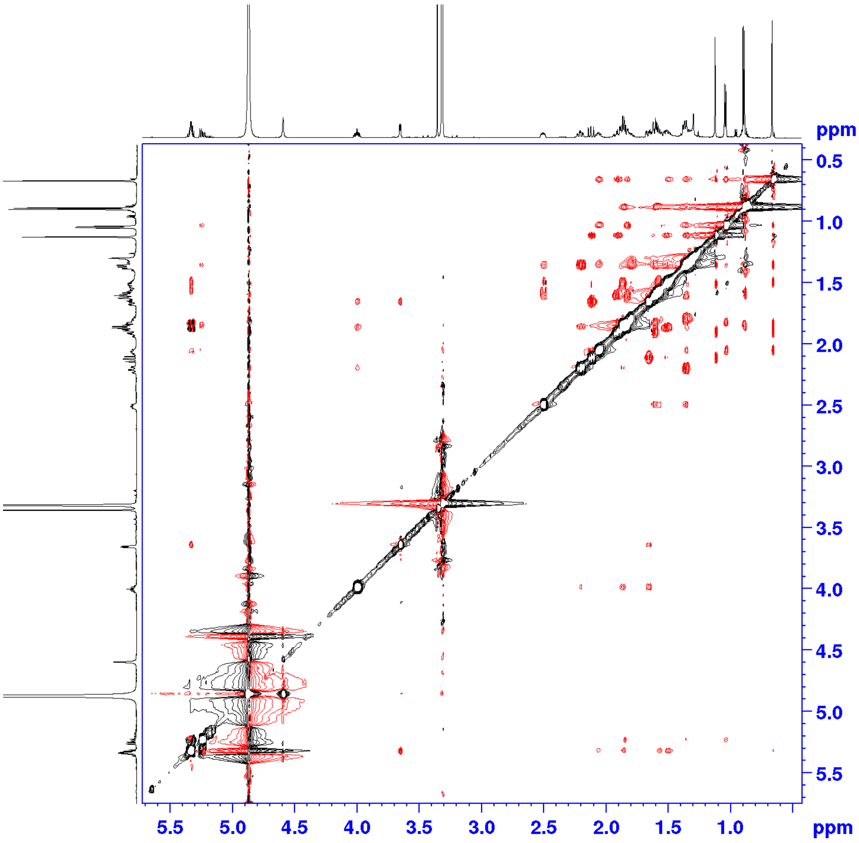

Figure S6. NOESY spectrum (600 MHz) of **1** in CD_3OD.

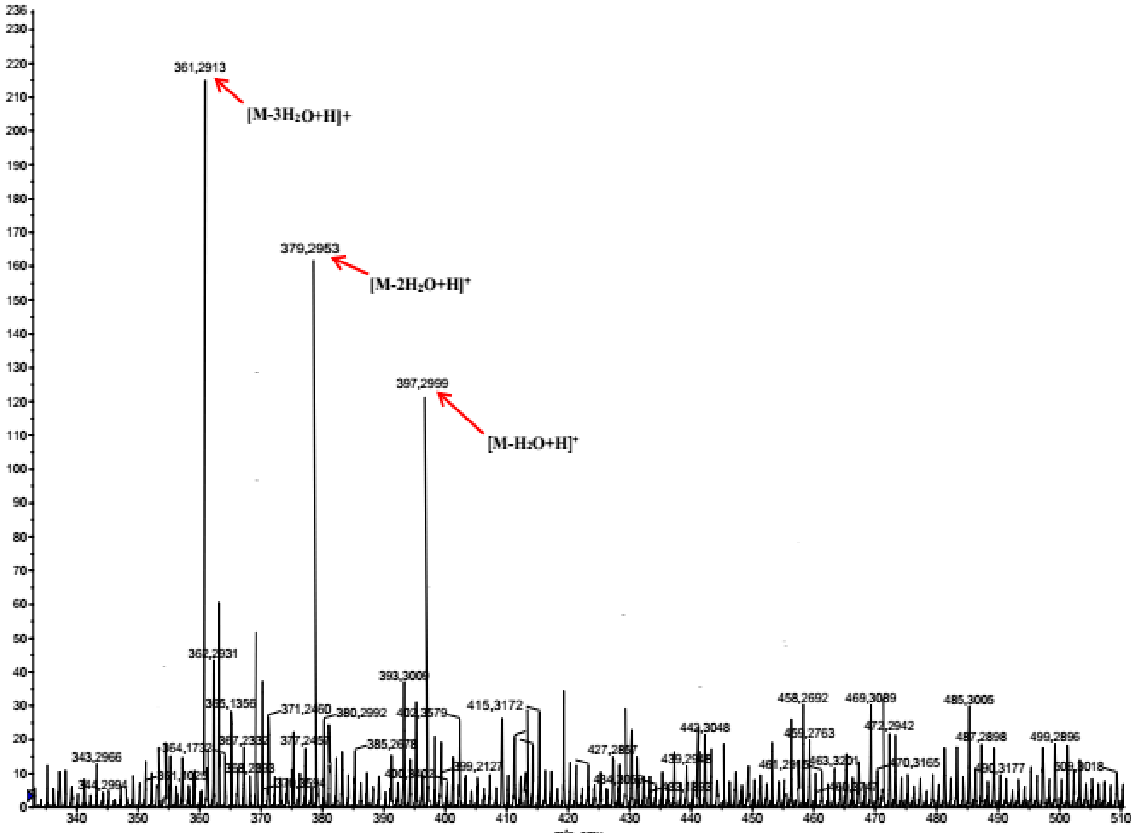

Figure S7. HR-ESI mass spectrum of **1**.

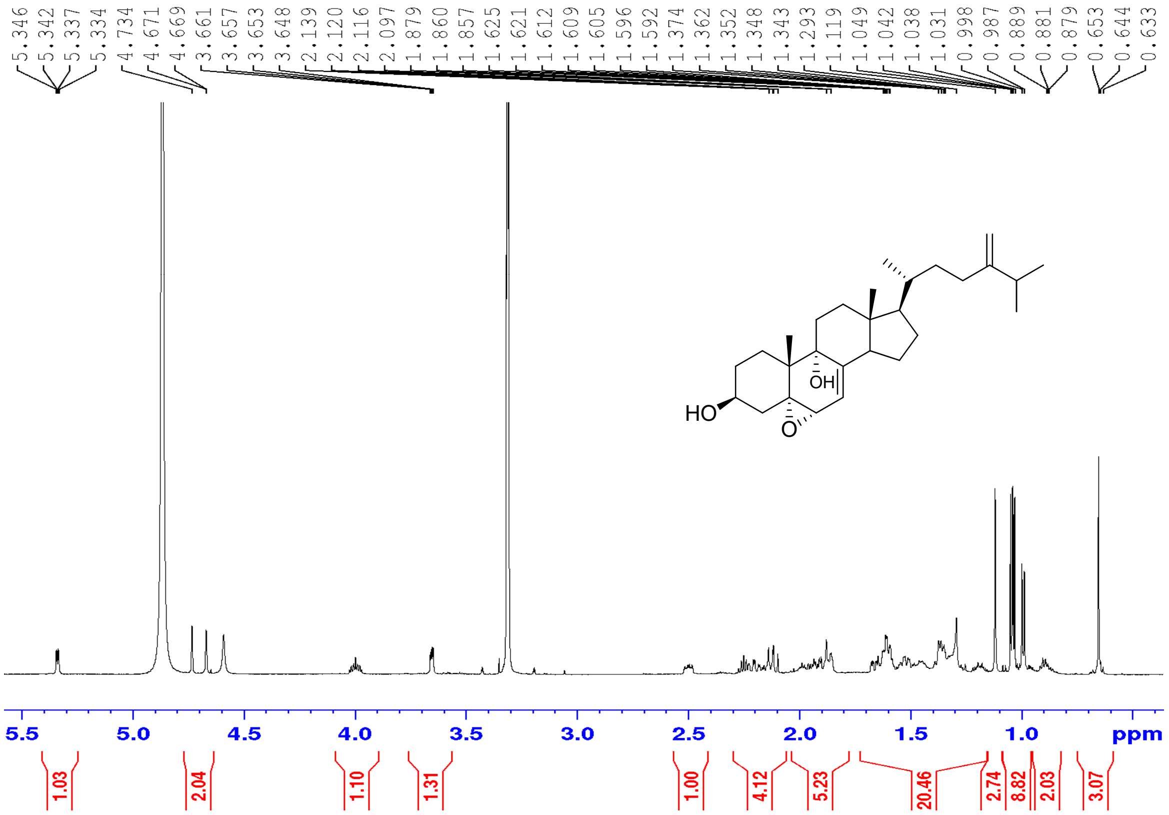

Figure S8. ^{1}H NMR spectrum (600 MHz) of 5α,6α-Epoxycholesta-7,24(28)-dien-3β,9α-diol (**2**) in CD3OD.

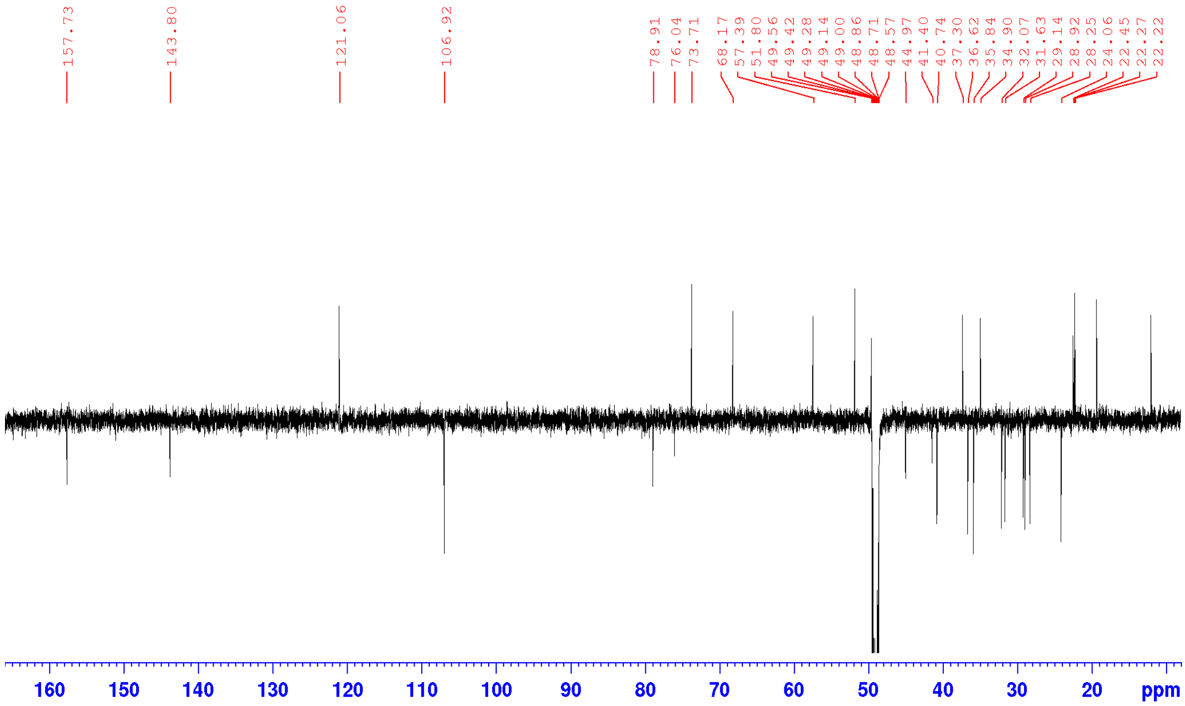

Figure S9. DEPT spectrum (150.9 MHz) of **2** in CD3OD.

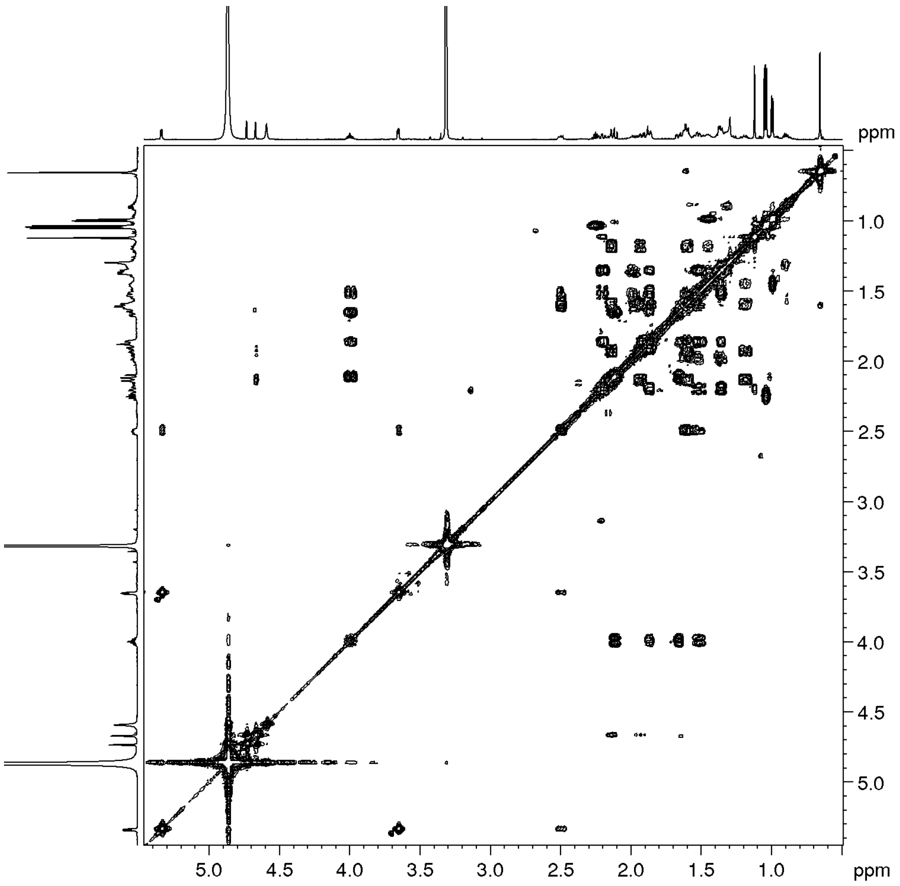

Figure S10. COSY spectrum (600 MHz) of **2** in CD_3OD.

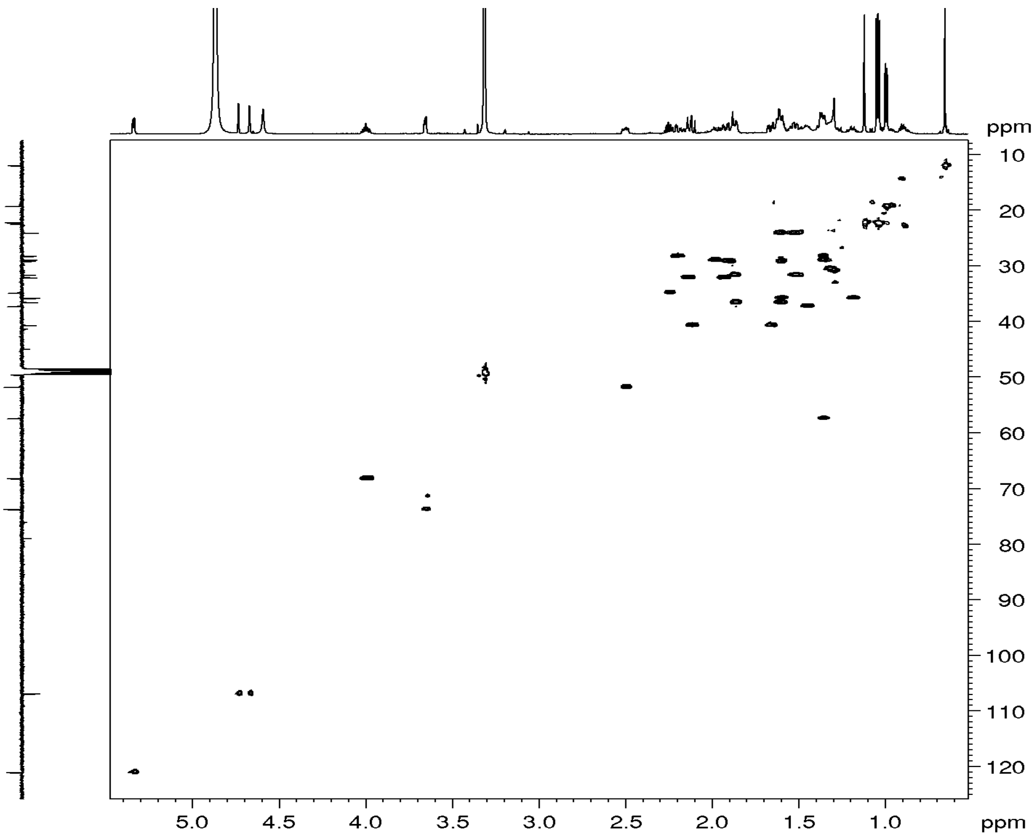

Figure S11. HSQC spectrum (600 MHz) of **2** in CD_3OD.

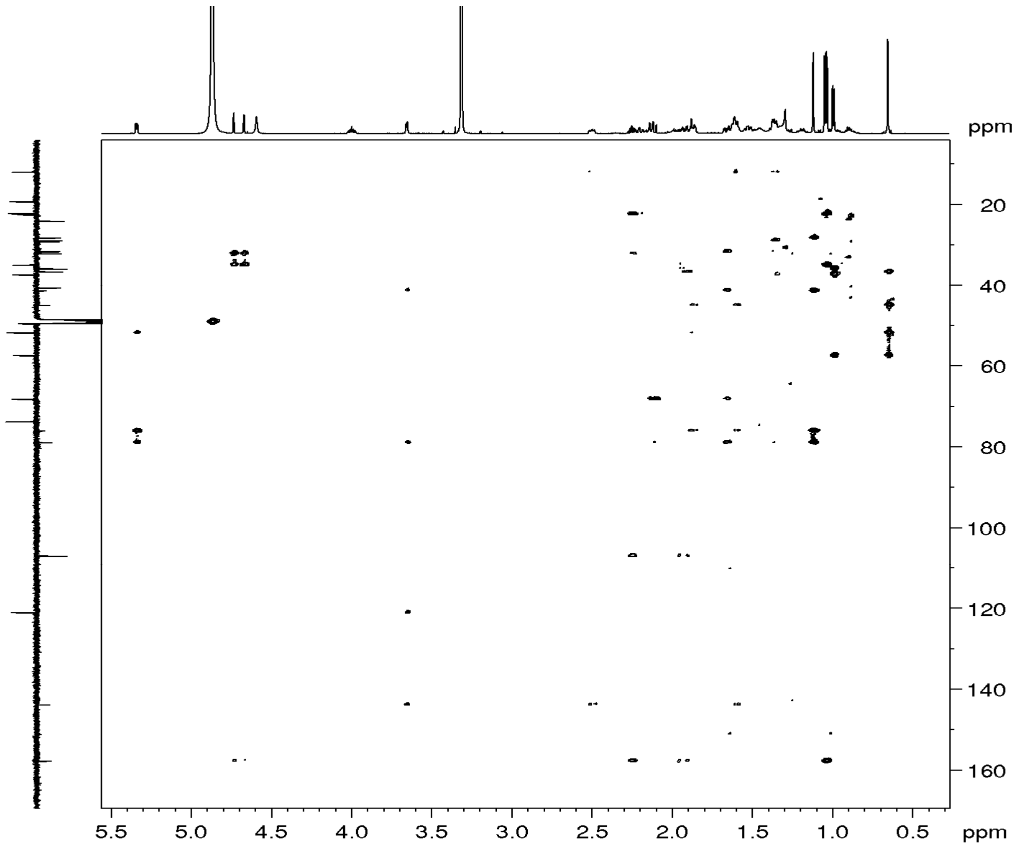

Figure S12. HMBC spectrum (600 MHz) of **2** in CD_3OD.

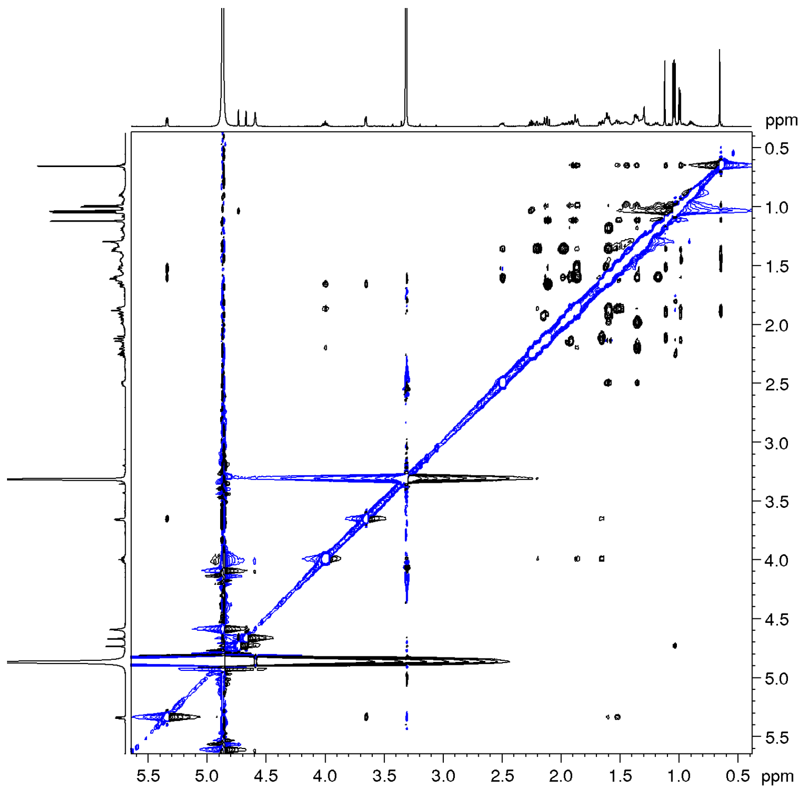

Figure S13. NOESY spectrum (600 MHz) of **2** in CD_3OD.

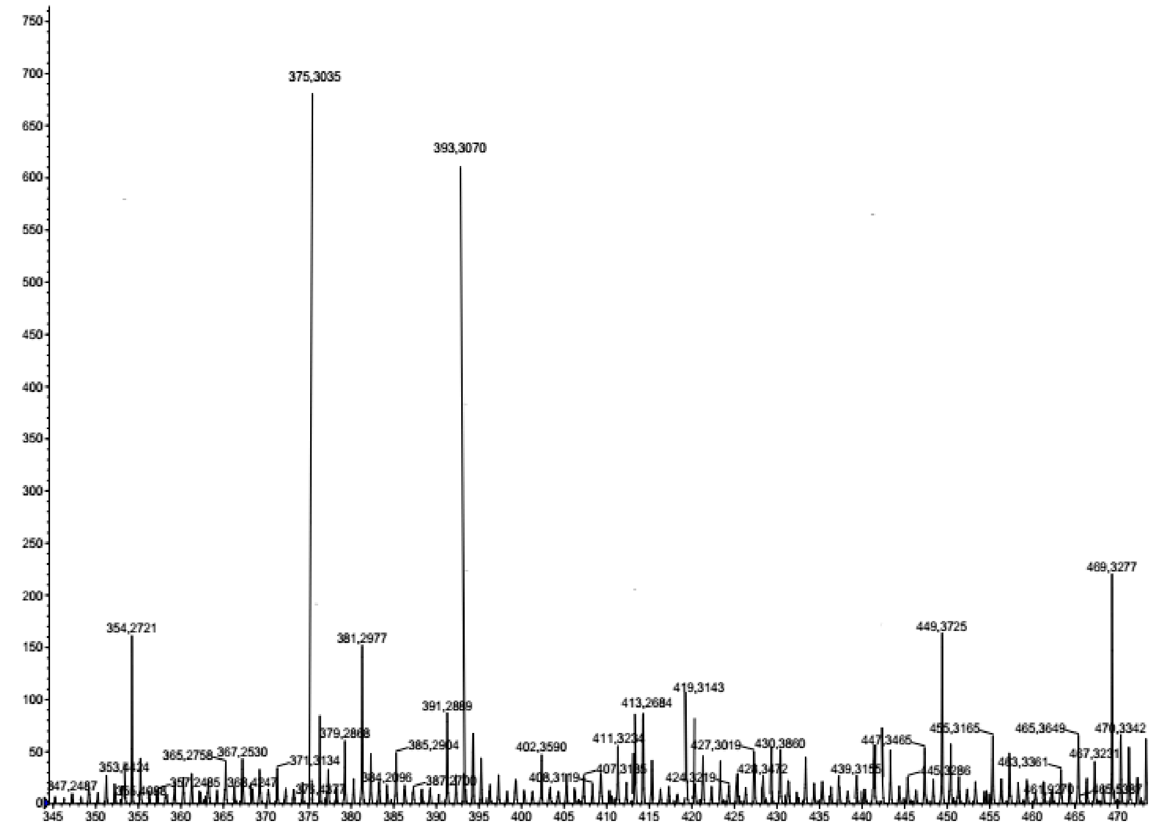

Figure S14. HR-ESI mass spectrum of **2**.

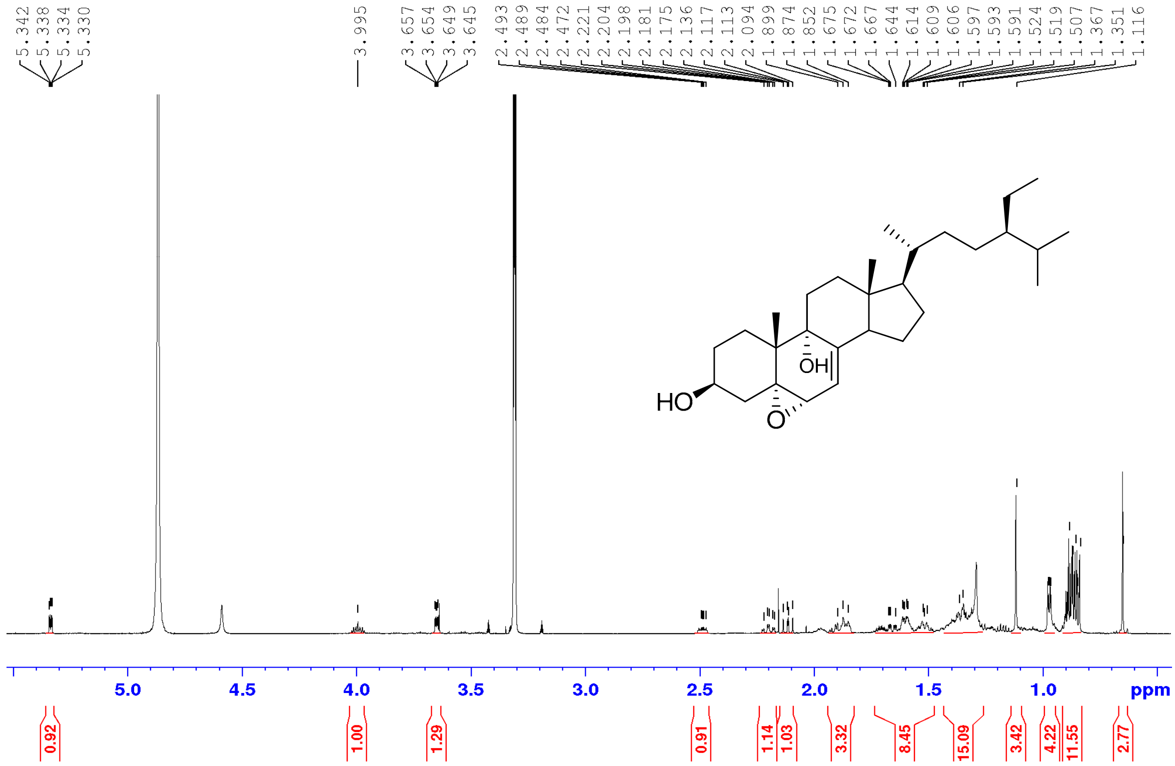

Figure S15. ^{1}H NMR spectrum (600 MHz) of (24*R*)-5α,6α-Epoxy-24-ethyl-cholesta-7-en-3β,9α-diol (**3**) in CD_3OD.

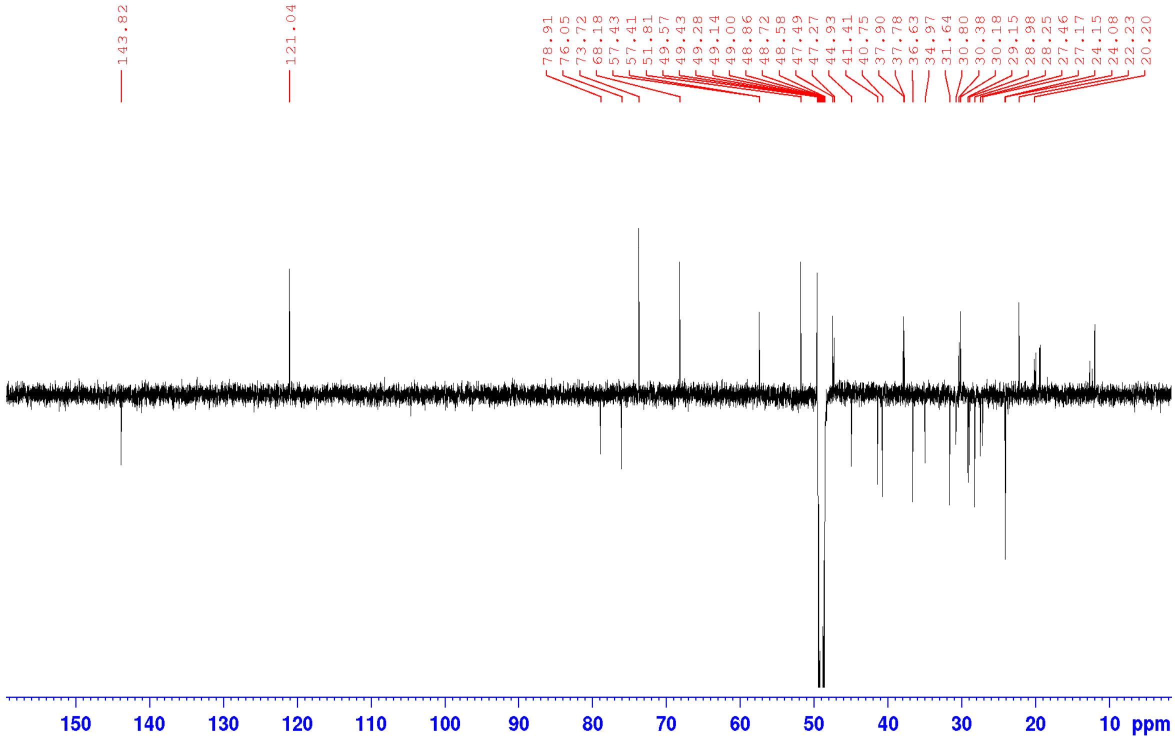

Figure S16. DEPT spectrum (150.9 MHz) of **3** in CD_3OD.

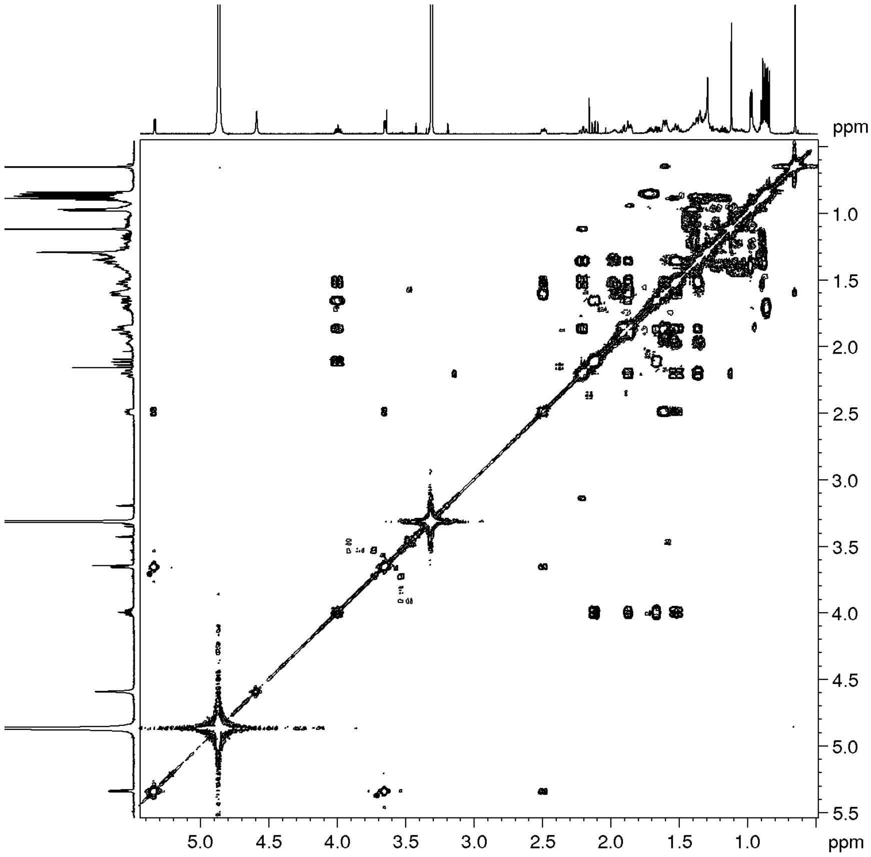

Figure S17. COSY spectrum (600 MHz) of **3** in CD_3OD.

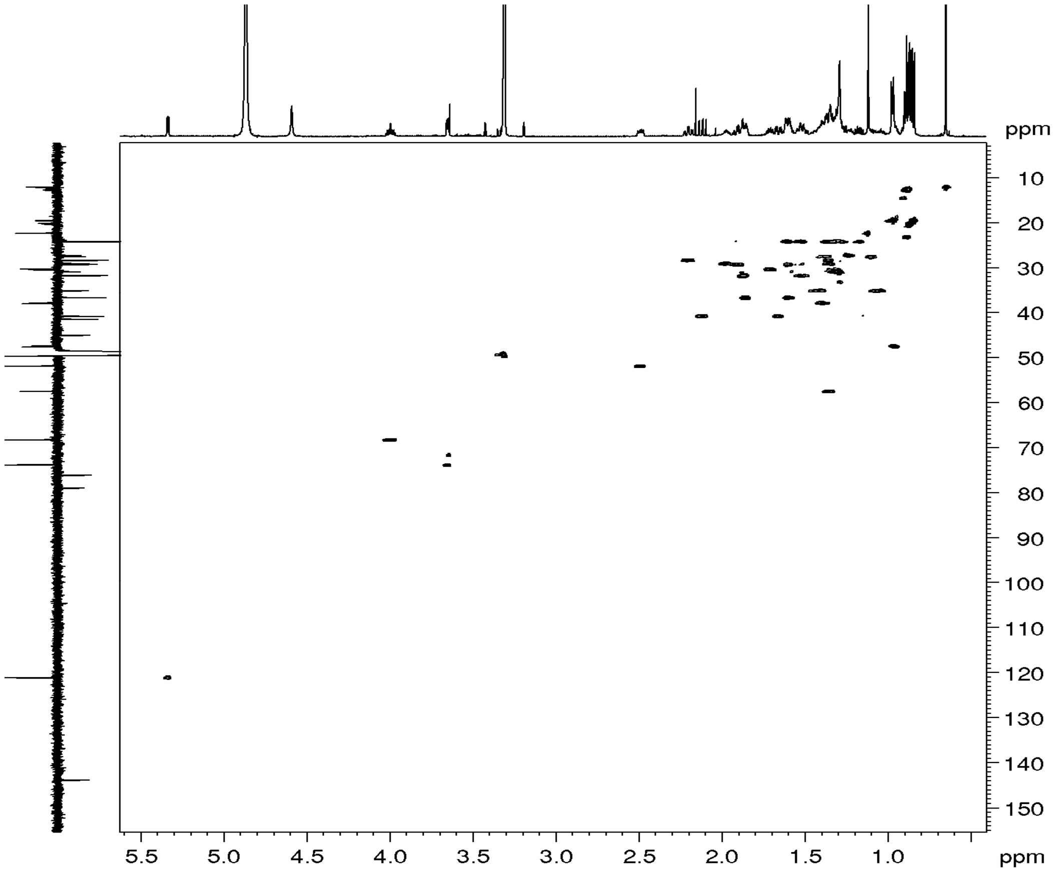

Figure S18. HSQC spectrum (600 MHz) of **3** in CD_3OD.

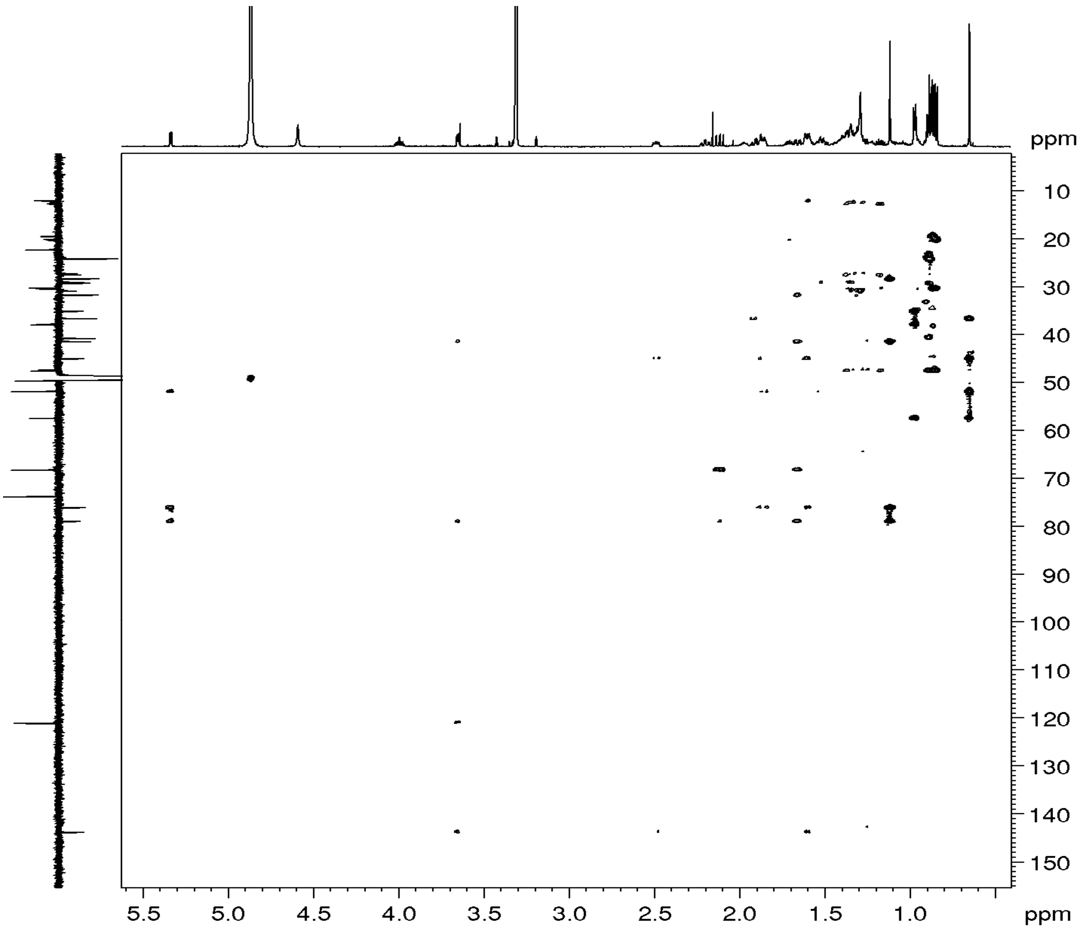

Figure S19. HMBC spectrum (600 MHz) of **3** in CD3OD.

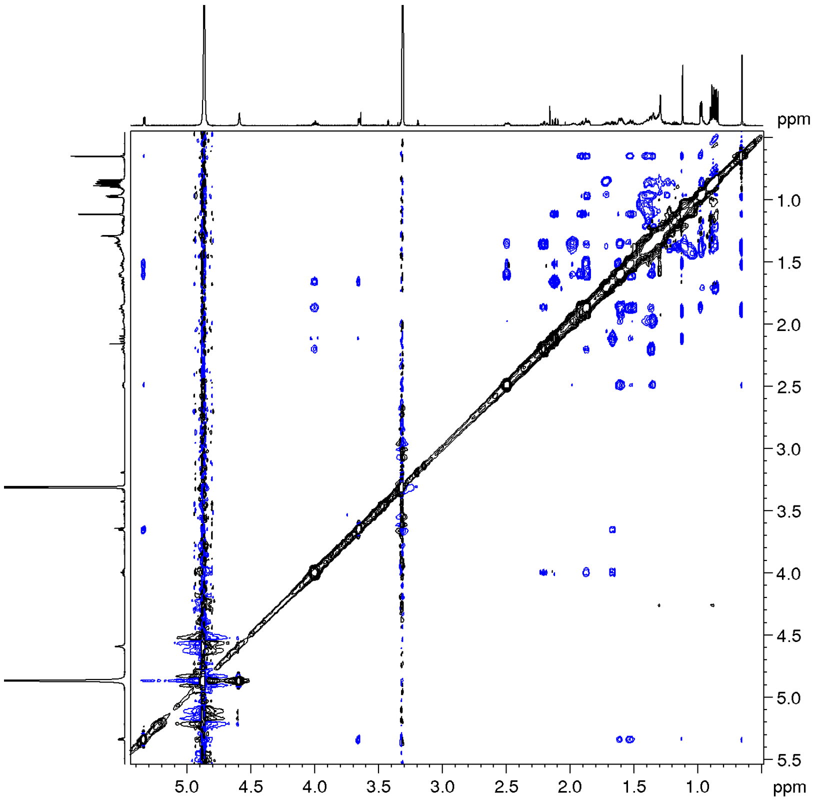

Figure S20. NOESY spectrum (600 MHz) of **3** in CD3OD.

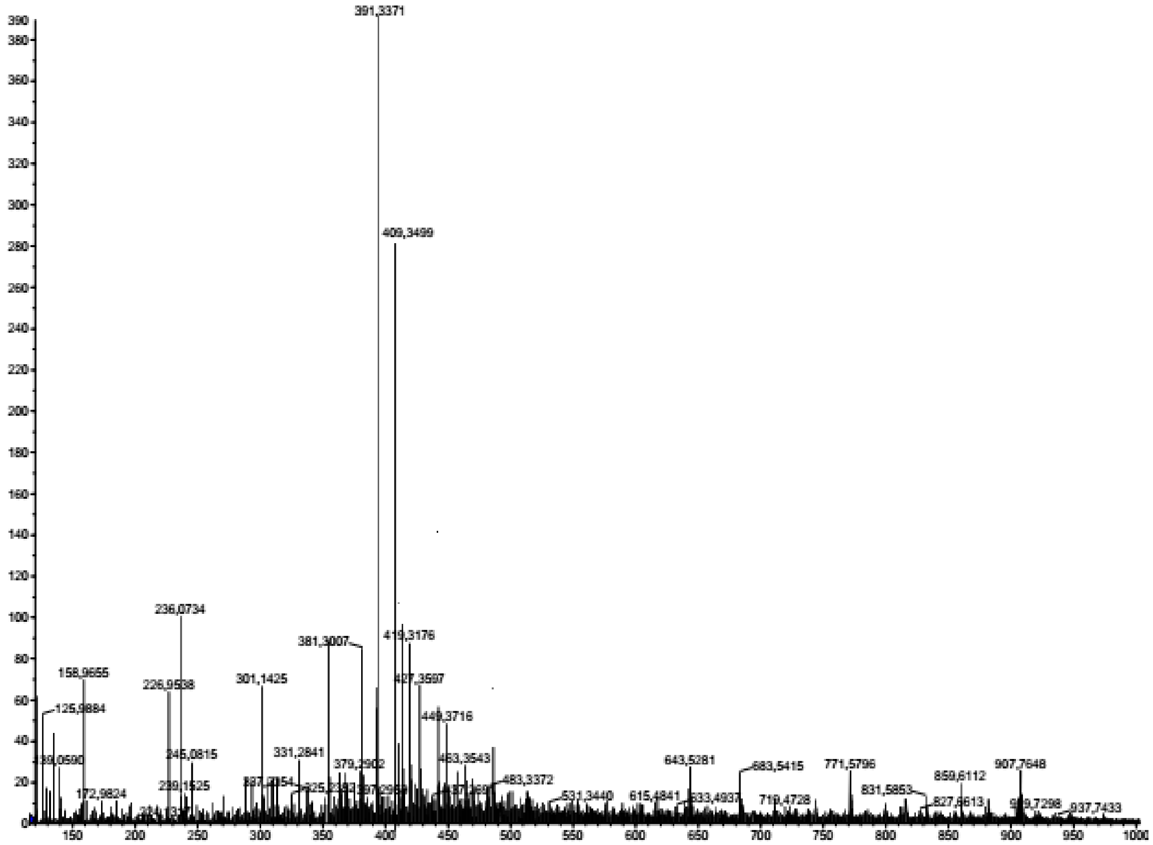

Figure S21. HR-ESI mass spectrum of **3**.

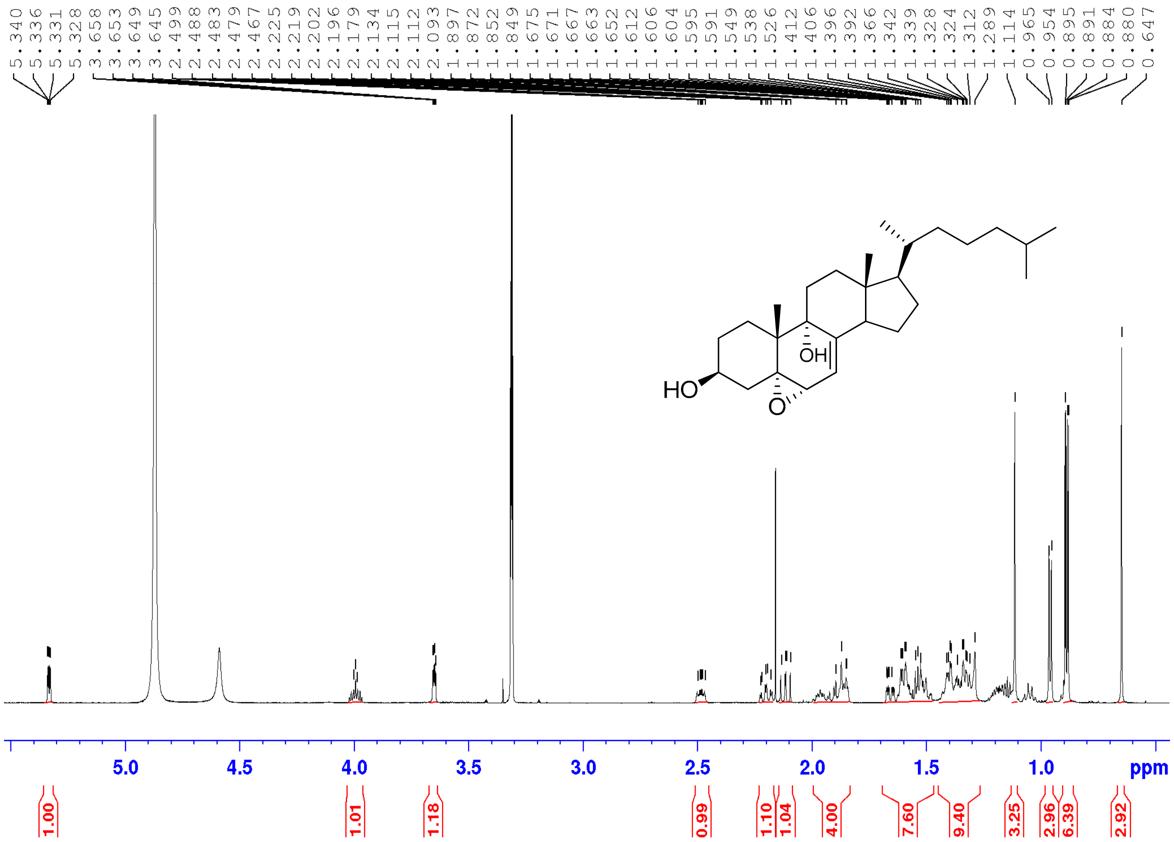

Figure S22. ^{1}H NMR spectrum (600 MHz) of 5*α*,6*α* -Epoxycholesta-7-en-3β,9α-diol (**4**) in CD_3OD.

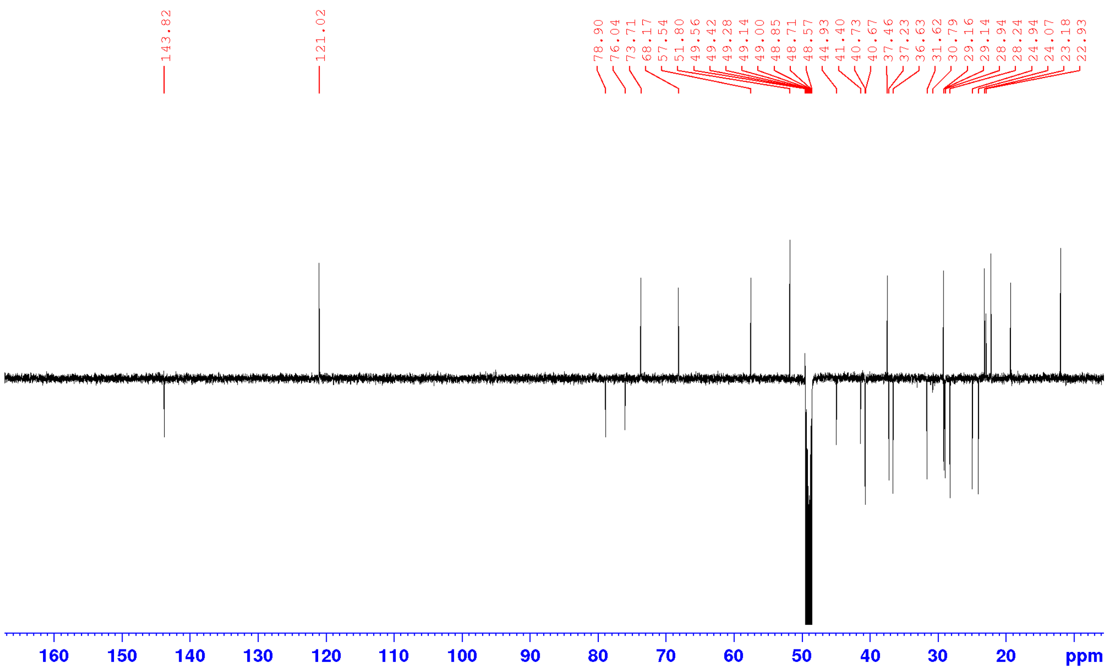

Figure S23. DEPT spectrum (150.9 MHz) of **4** in CD_3OD.

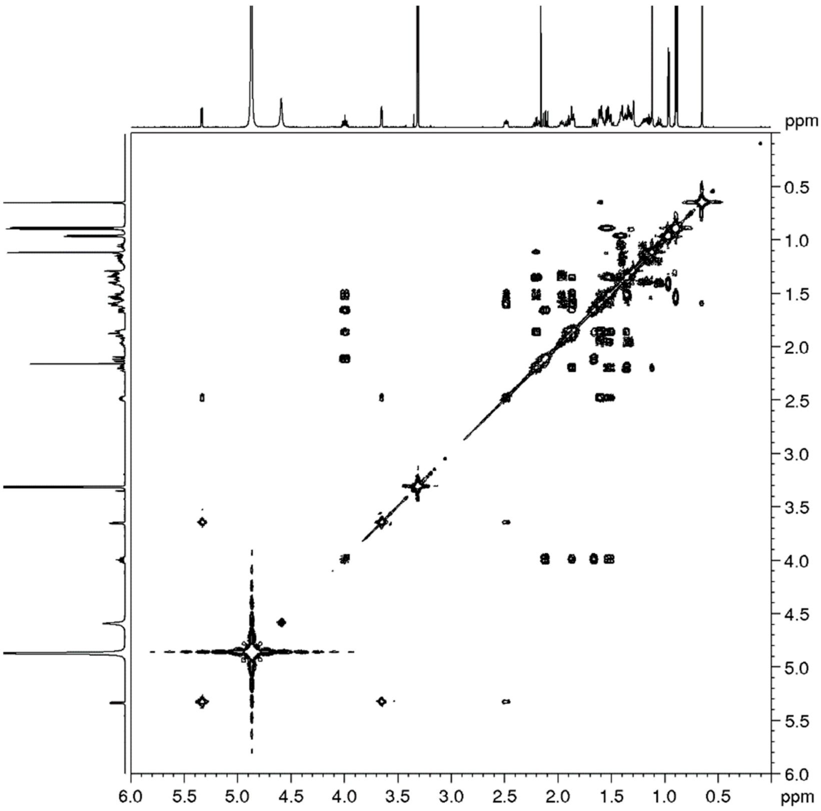

Figure S24. COSY spectrum (600 MHz) of **4** in CD_3OD.

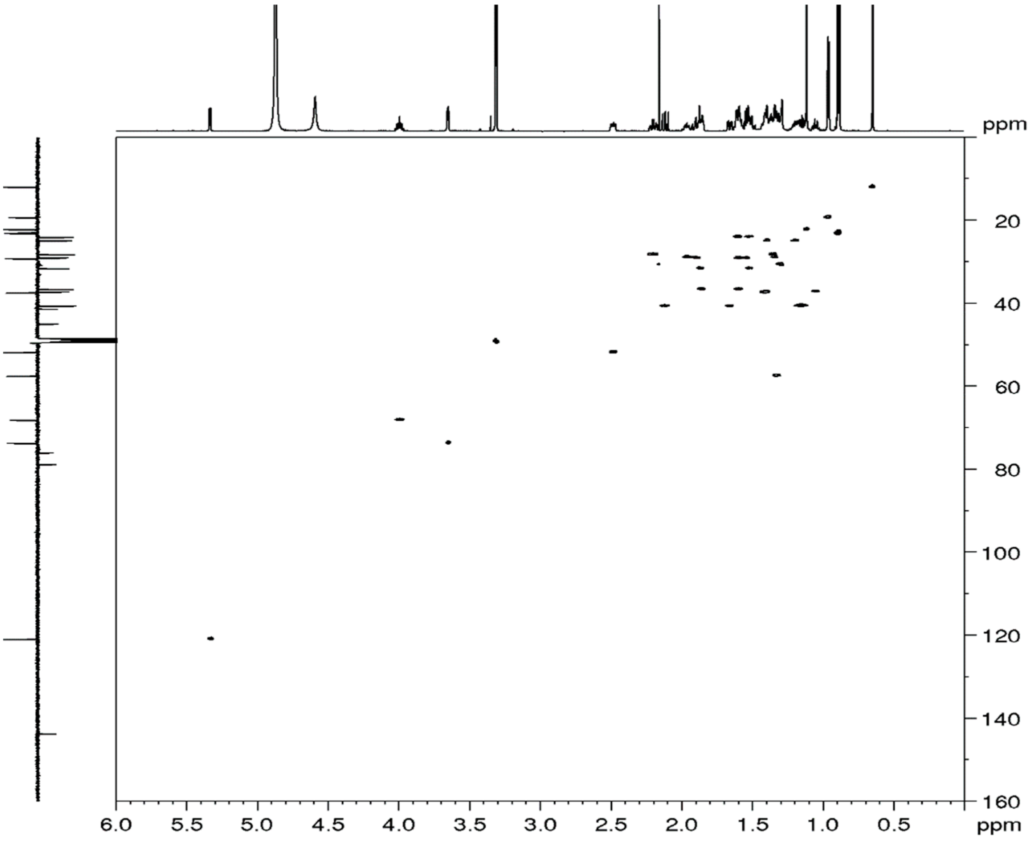

Figure S25. HSQC spectrum (600 MHz) of **4** in CD_3OD.

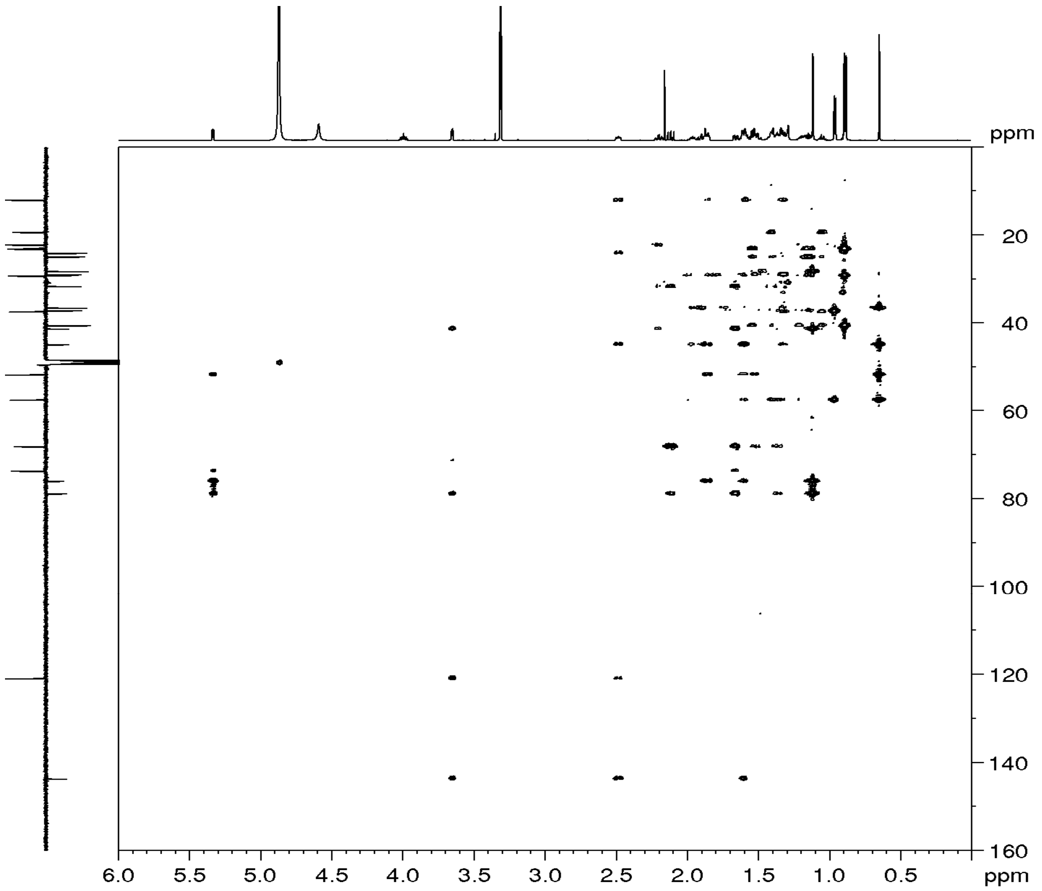

Figure S26. HMBC spectrum (600 MHz) of **4** in CD3OD.

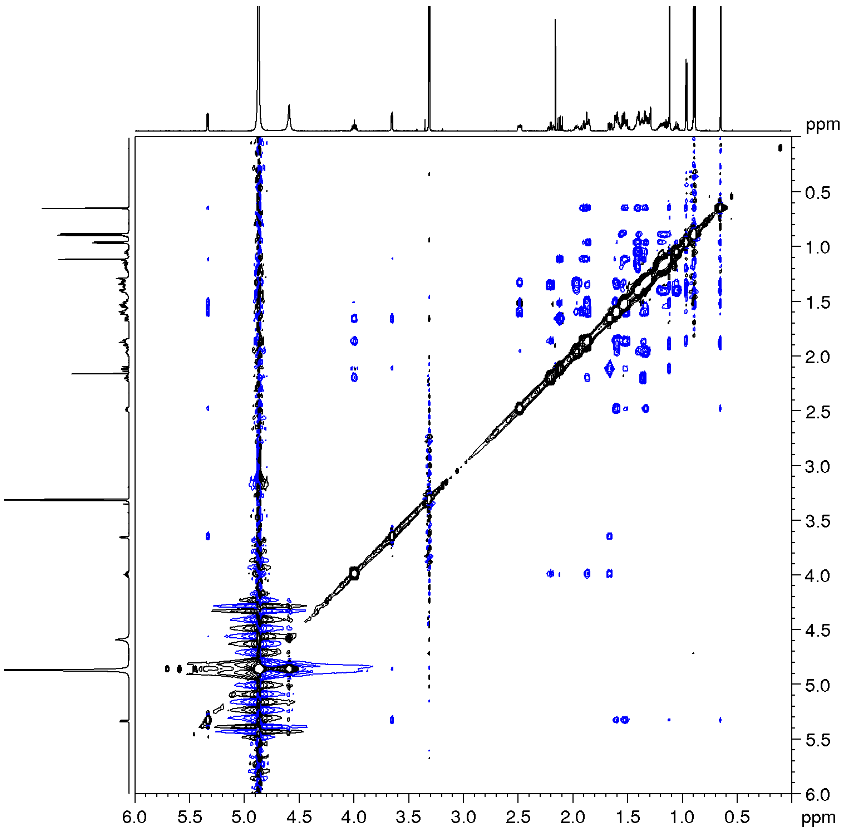

Figure S27. NOESY spectrum (600 MHz) of **4** in CD3OD.

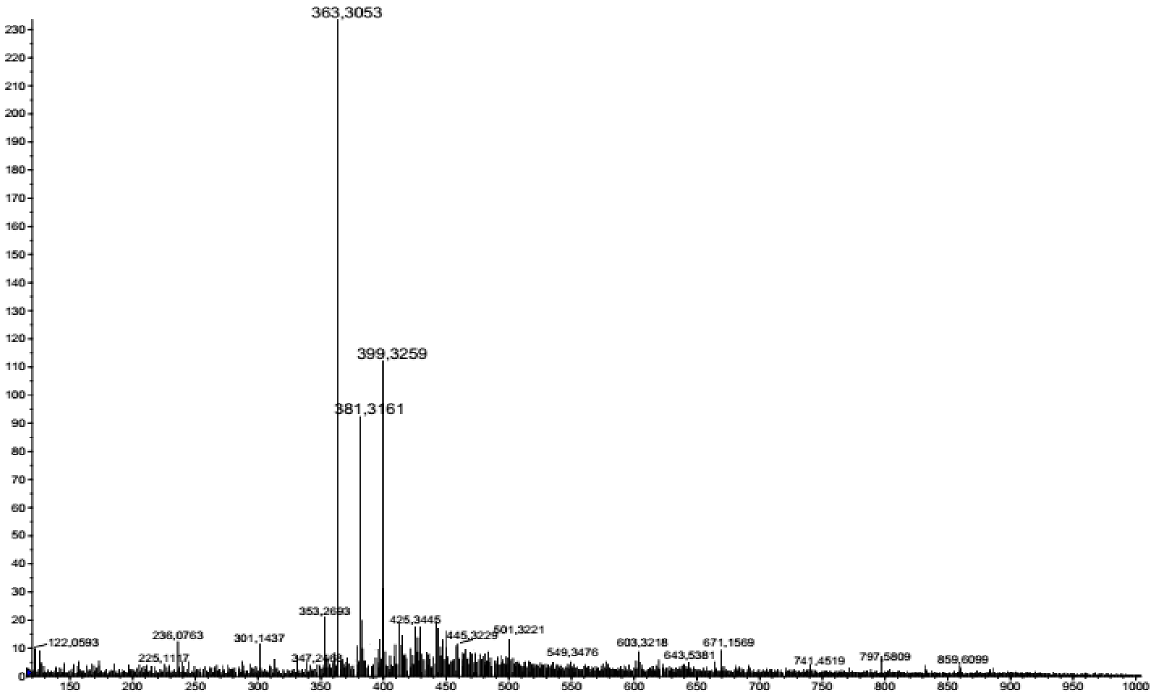

Figure S28. HR-ESI mass spectrum of **4**.

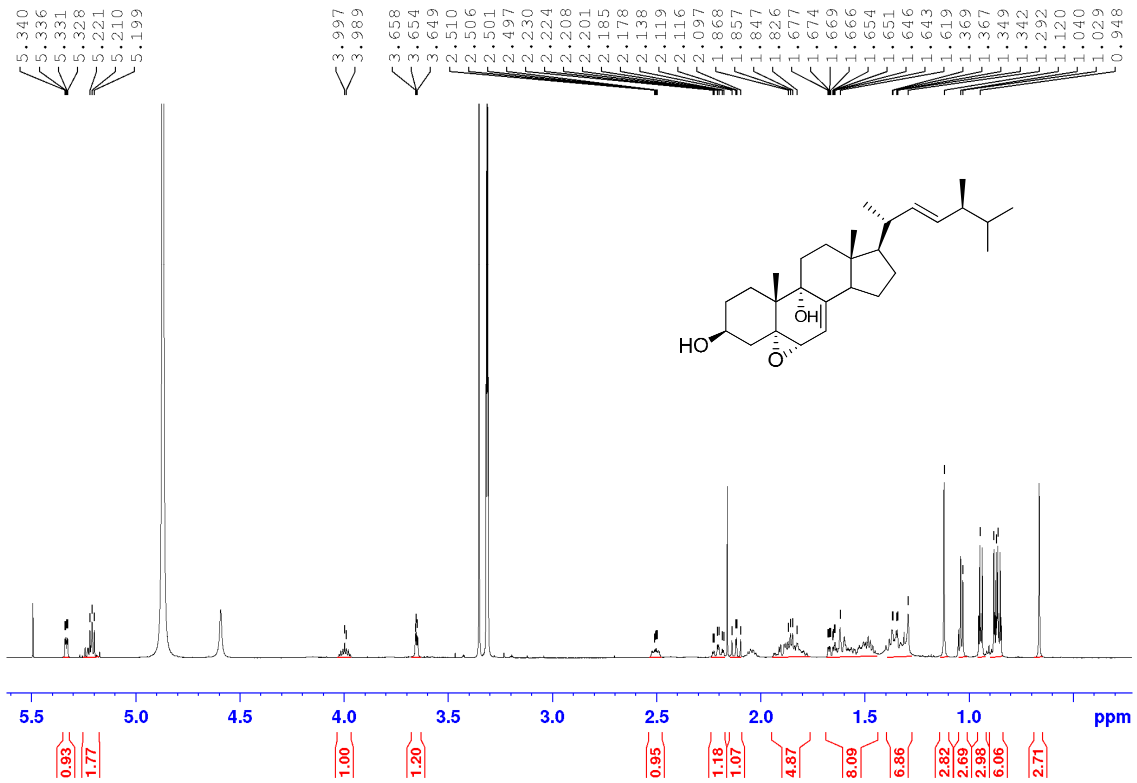

Figure S29. ^{1}H NMR spectrum (600 MHz) of (24*S*)-5*α*,6*α*-Epoxyergosta-7,22-dien-3β,9α-diol (**5**) in CD_3OD.

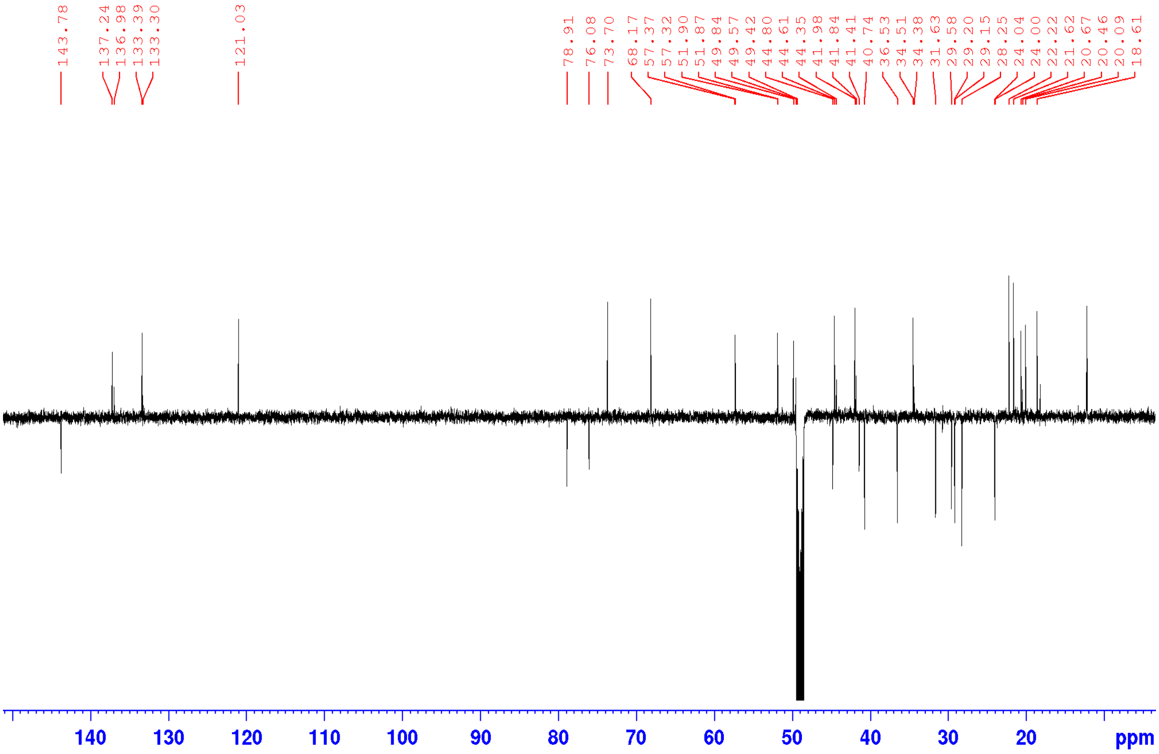

Figure S30. DEPT spectrum (150.9 MHz) of **5** in CD_3OD.

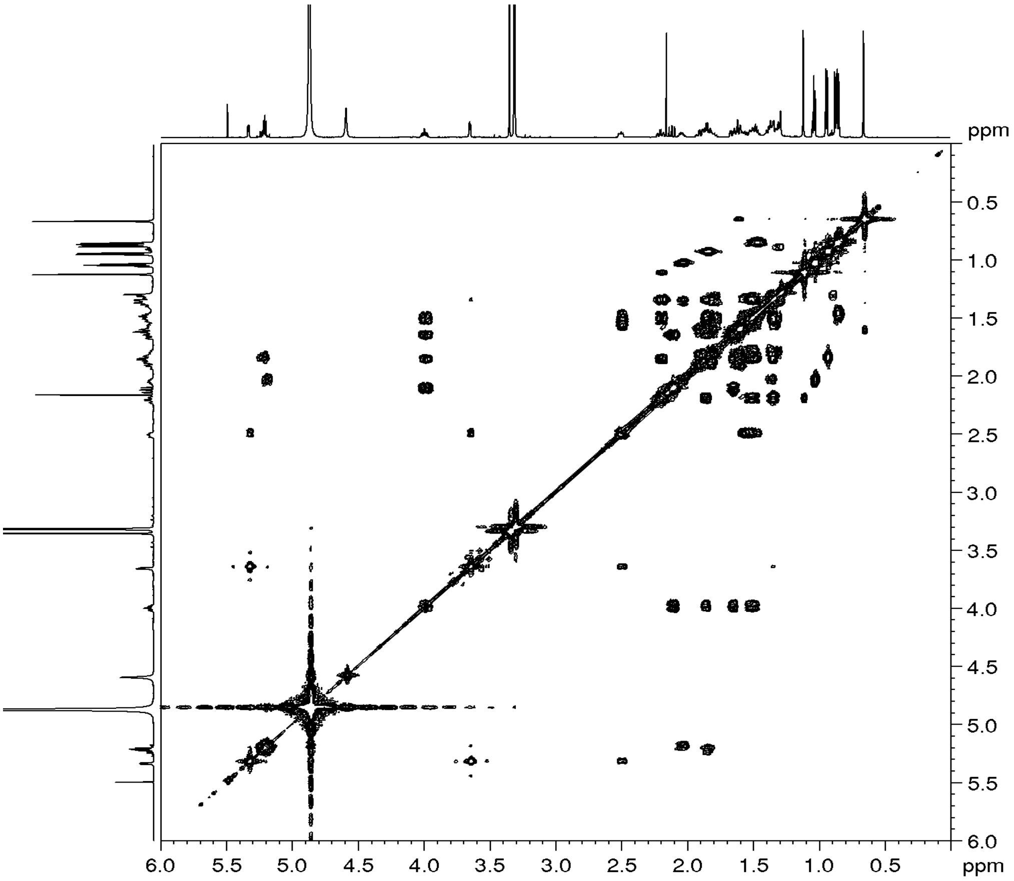

Figure S31. COSY spectrum (600 MHz) of **5** in CD_3OD.

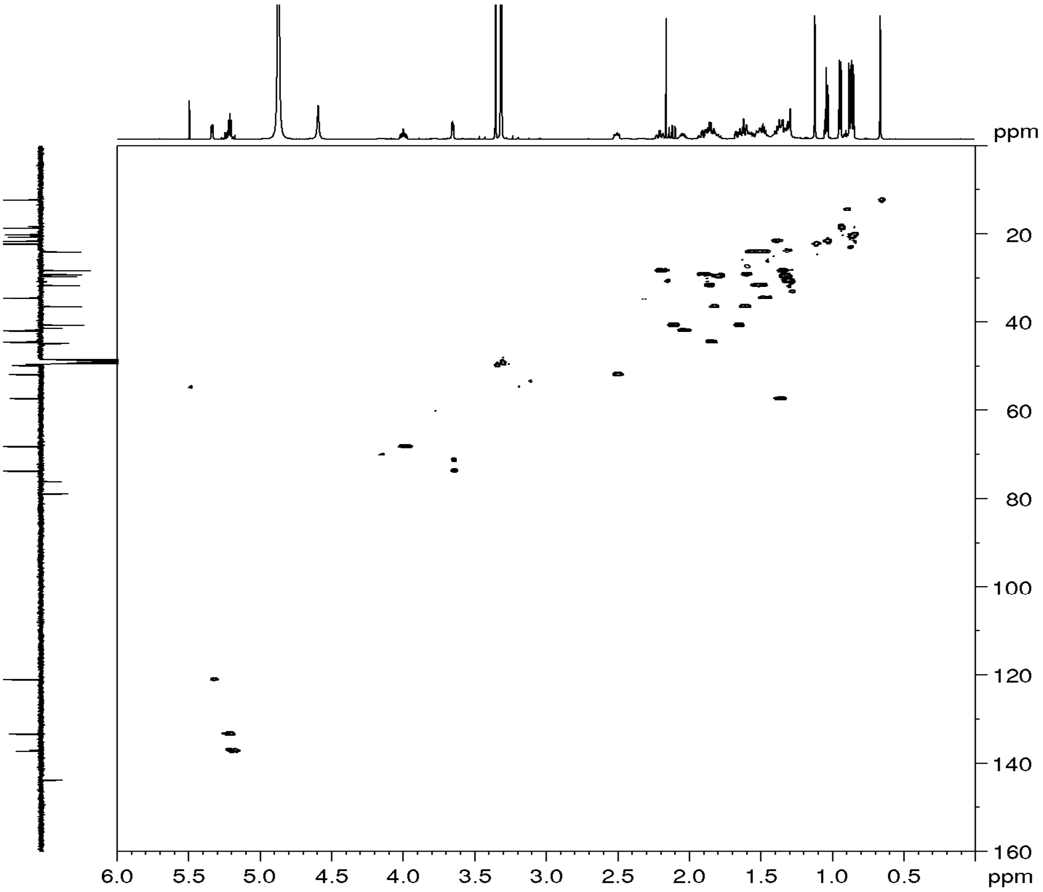

Figure S32. HSQC spectrum (600 MHz) of **5** in CD_3OD.

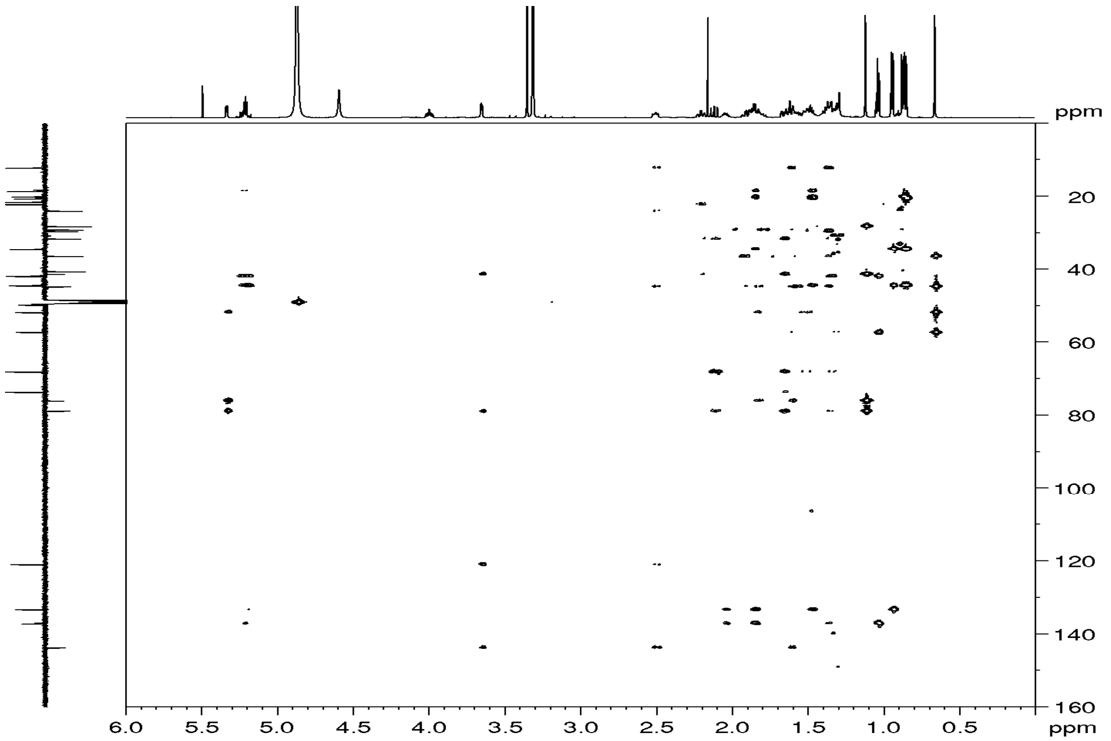

Figure S33. HMBC spectrum (600 MHz) of **5** in CD_3OD.

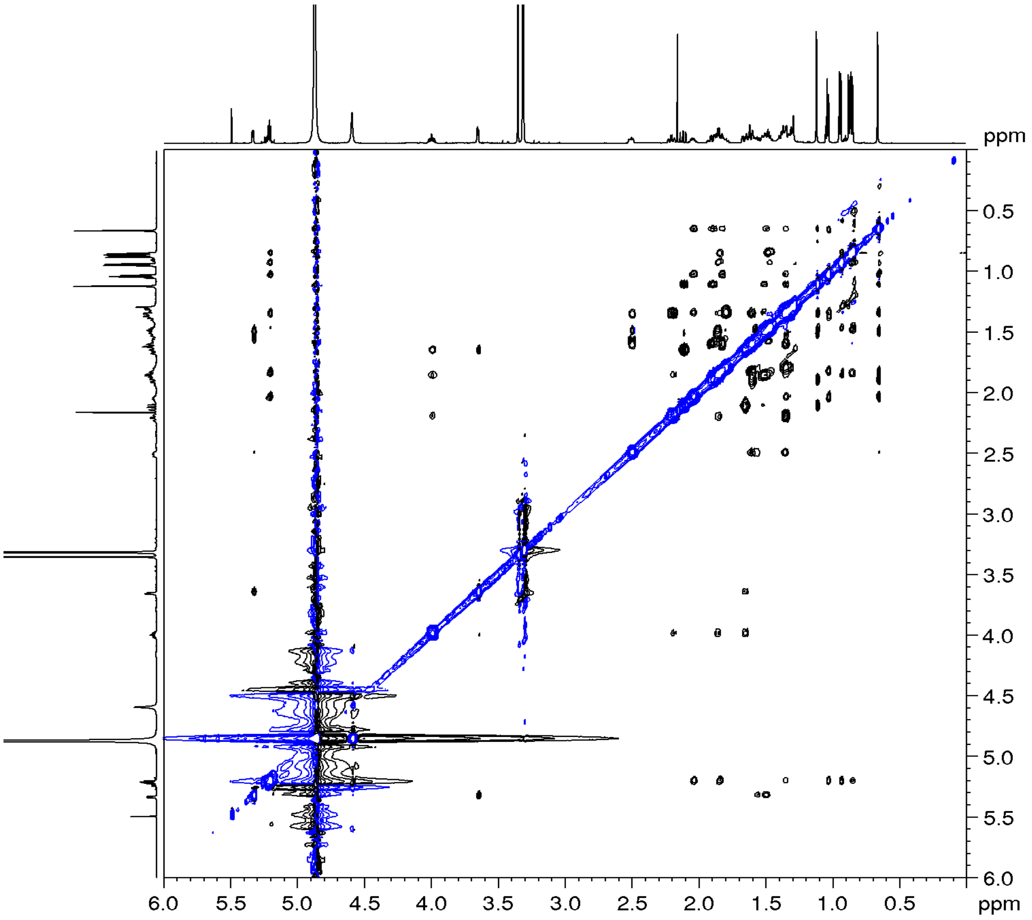

Figure S34. NOESY spectrum (600 MHz) of **5** in CD_3OD.

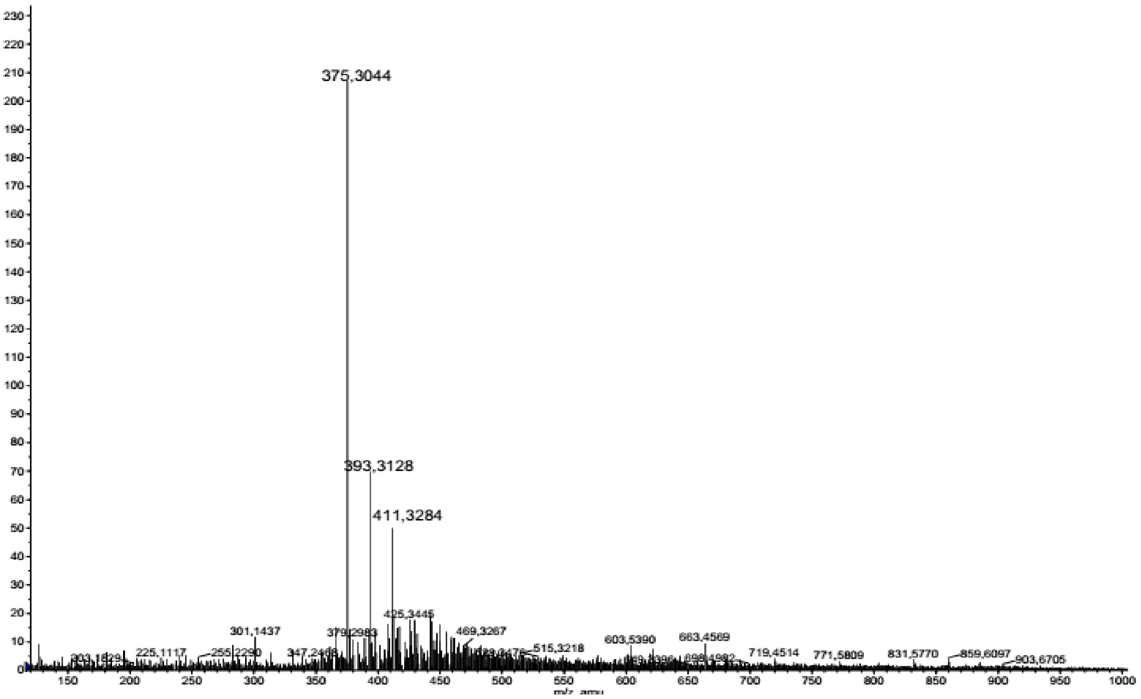

Figure S35. HR-ESI mass spectrum of **5**.

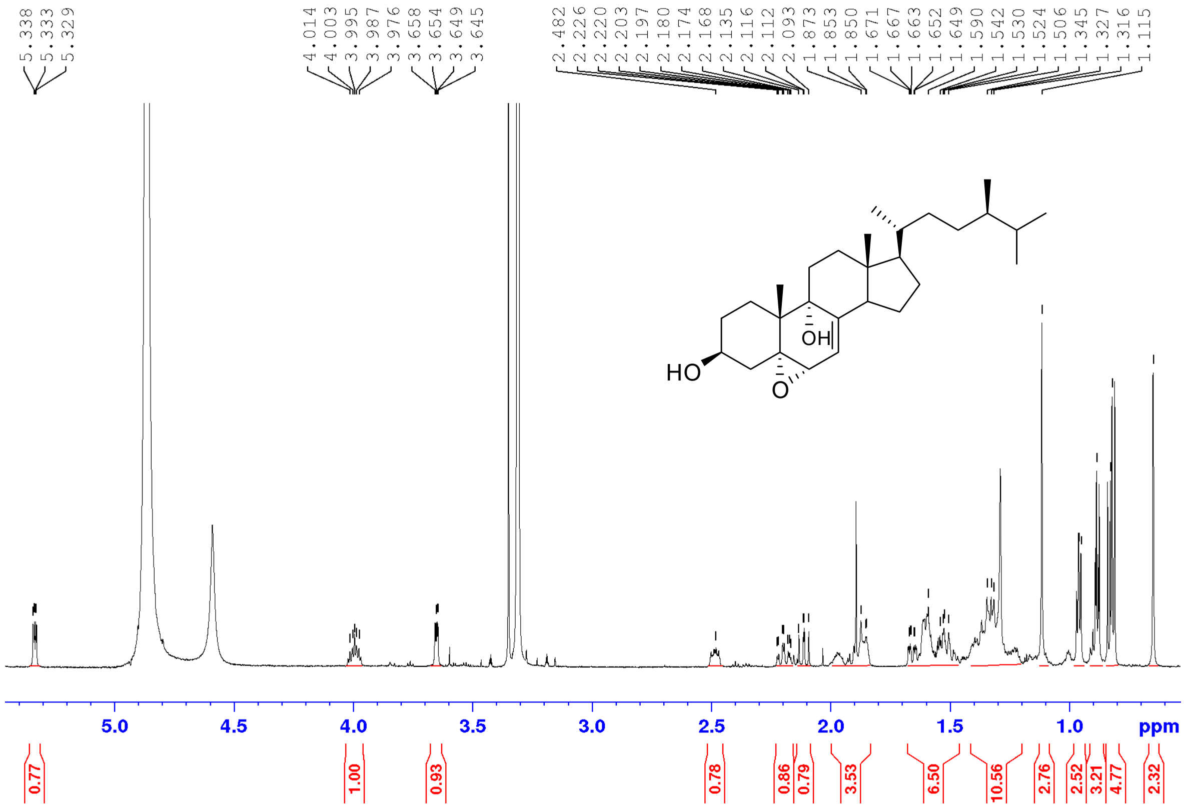

Figure S36. ^{1}H NMR spectrum (600 MHz) of (24*R*)-5α,6α-Epoxy-24-methyl-cholesta-7-en-3β,9α-diol (**6**) in CD_3OD.

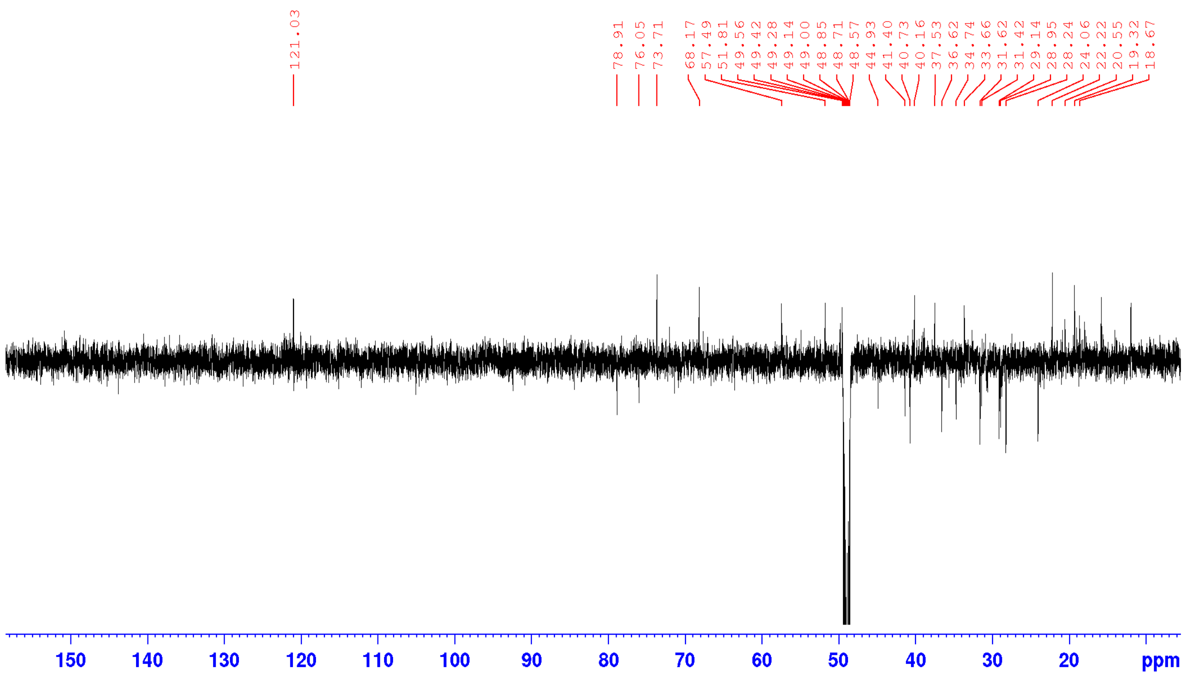

Figure S37. DEPT spectrum (150.9 MHz) of **6** in CD_3OD.

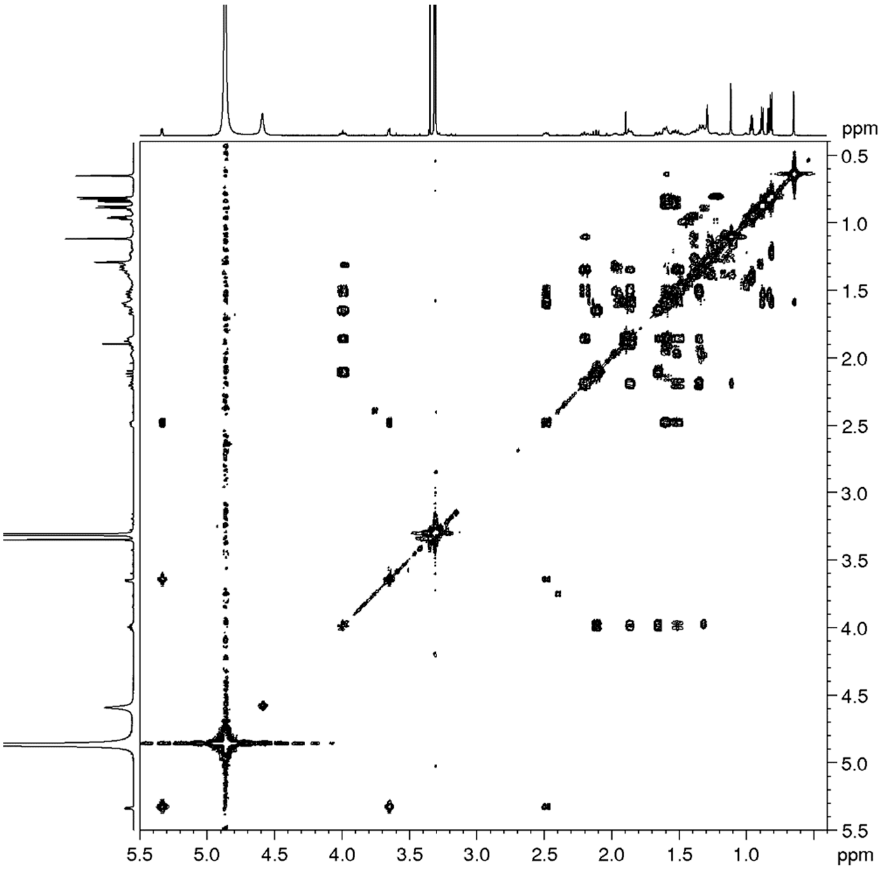

Figure S38. COSY spectrum (600 MHz) of **6** in CD_3OD.

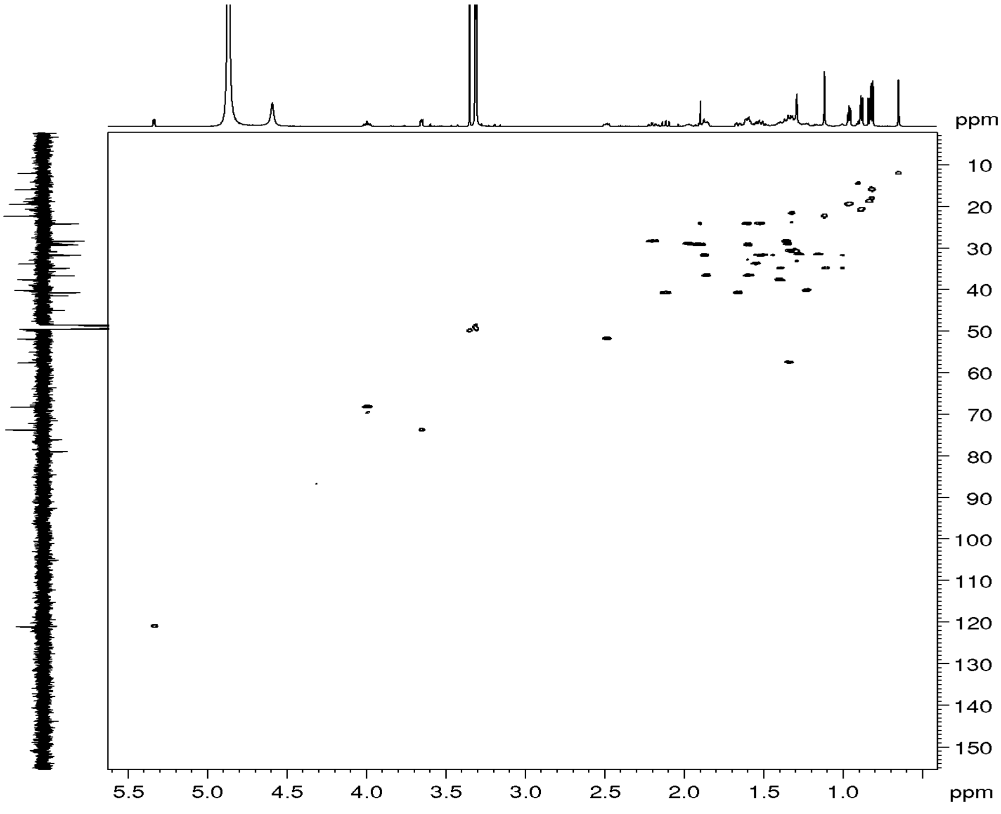

Figure S39. HSQC spectrum (600 MHz) of **6** in CD_3OD.

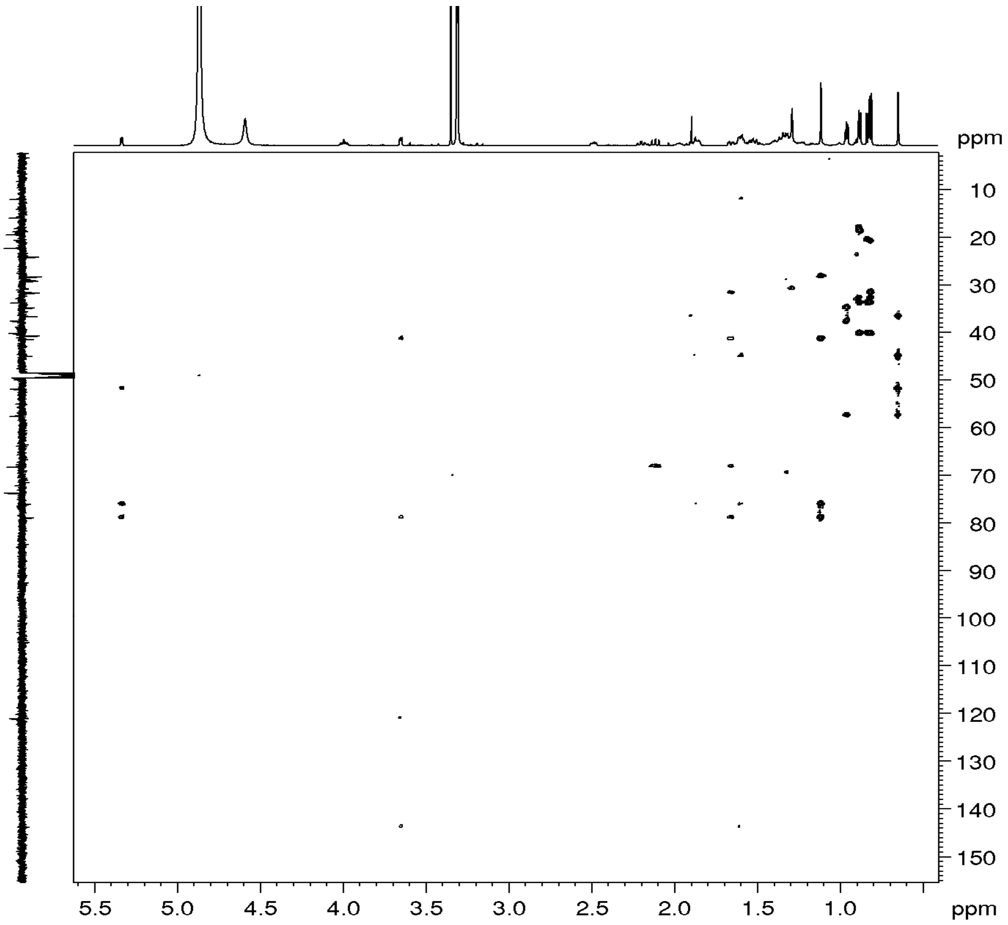

Figure S40. HMBC spectrum (600 MHz) of **6** in CD_3OD.

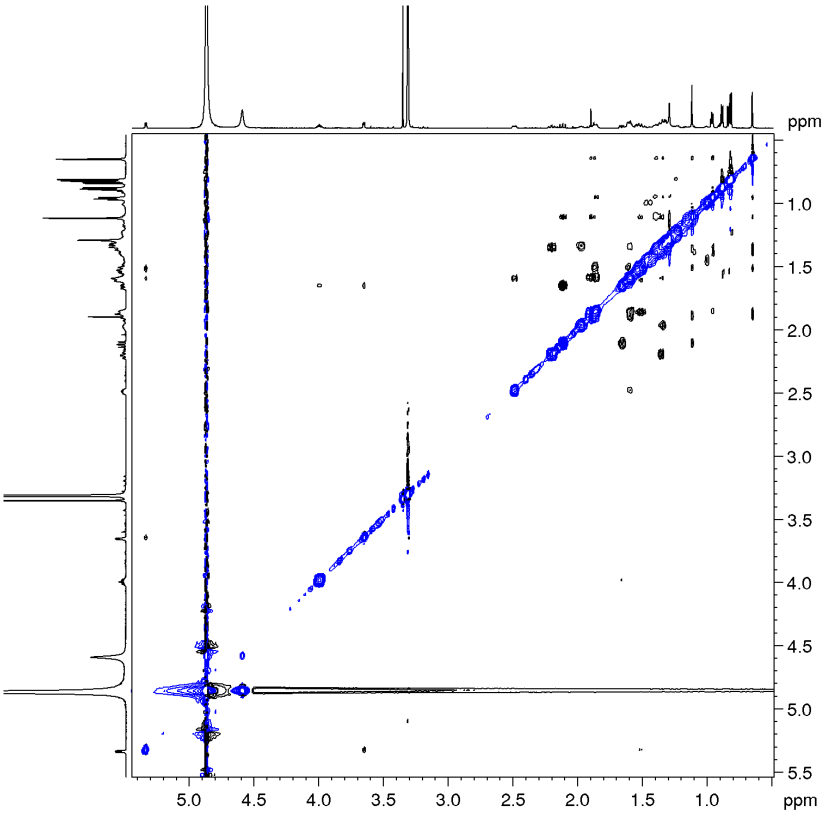

Figure S41. NOESY spectrum (600 MHz) of 6 in CD_3OD.

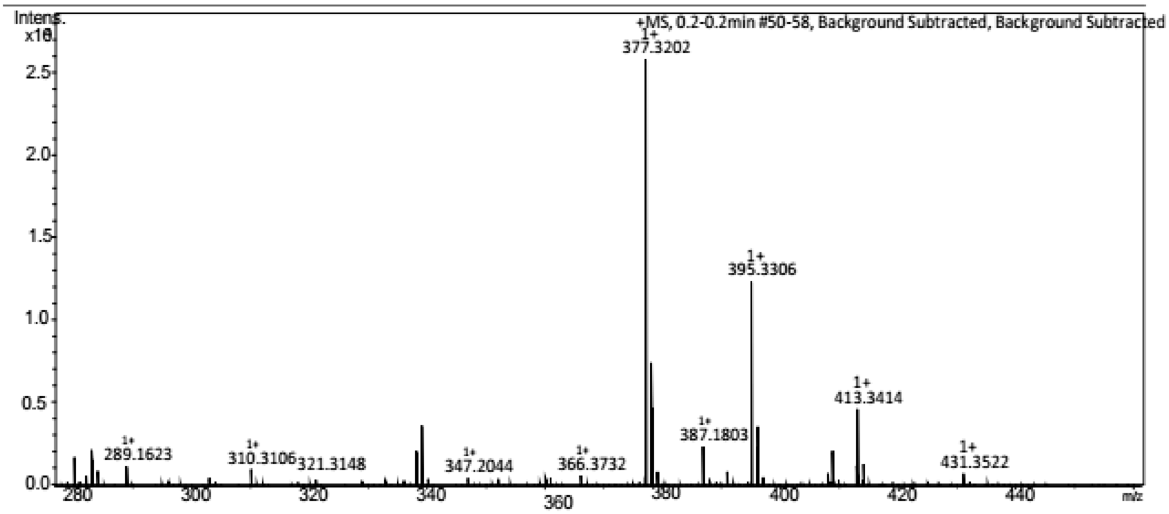

Figure S42. HR-ESI mass spectrum of **6**.

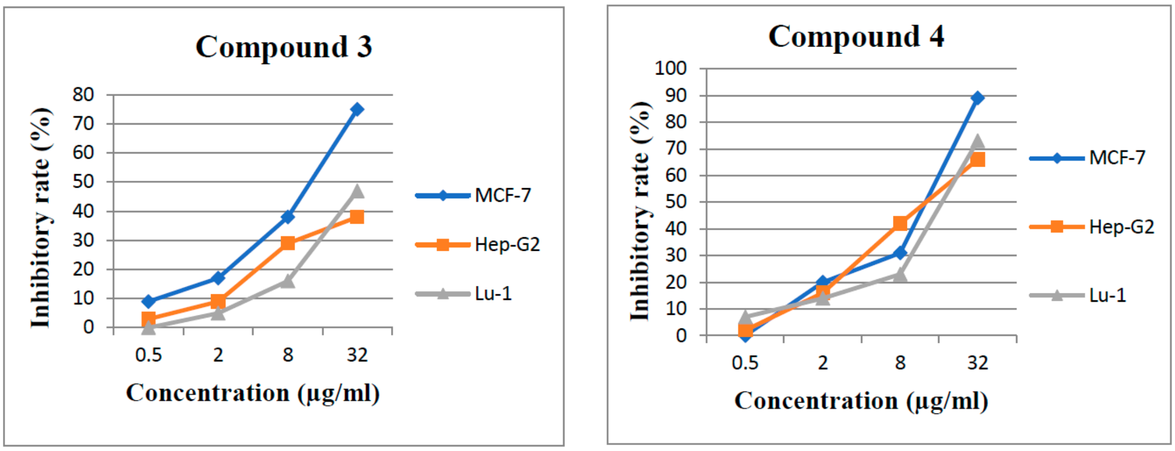

Figure S43. Growth inhibition curves of the active compounds **3** and **4** against three human cancer cell lines.
